# *Adnp*-mutant mice with cognitive inflexibility, CaMKIIα hyperactivity, and synaptic plasticity deficits

**DOI:** 10.1038/s41380-023-02129-5

**Published:** 2023-06-26

**Authors:** Heejin Cho, Taesun Yoo, Heera Moon, Hyojin Kang, Yeji Yang, MinSoung Kang, Esther Yang, Dowoon Lee, Daehee Hwang, Hyun Kim, Doyoun Kim, Jin Young Kim, Eunjoon Kim

**Affiliations:** 1 Department of Biological Sciences, Korea Advanced Institute for Science and Technology (KAIST), Daejeon, 34141 Korea; 2 Center for Synaptic Brain Dysfunctions, Institute for Basic Science (IBS), Daejeon, 34141 Korea; 3 Division of National Supercomputing, Korea Institute of Science and Technology Information, Daejeon, 34141 Korea; 4 Research Center for Bioconvergence Analysis, Korea Basic Science Institute, 162 Yeongudanjiro, Ochang, Cheongju, Chungbuk 28119 Korea; 5 Therapeutics & Biotechnology Division, Drug discovery platform research center, Korea Research Institute of Chemical Technology (KRICT), Daejeon, 34114 Korea; 6 Department of Anatomy and BK21 Graduate Program, Biomedical Sciences, College of Medicine, Korea University, Seoul, 02841 Korea; 7 School of Biological Sciences, Seoul National University, Seoul, 08826 Korea; 8 Medicinal Chemistry and Pharmacology, Korea University of Science and Technology (UST), Daejeon, 34113 Korea

**Keywords:** Neuroscience, Molecular biology

## Abstract

ADNP syndrome, involving the ADNP transcription factor of the SWI/SNF chromatin-remodeling complex, is characterized by developmental delay, intellectual disability, and autism spectrum disorders (ASD). Although *Adnp*-haploinsufficient (Adnp-HT) mice display various phenotypic deficits, whether these mice display abnormal synaptic functions remain poorly understood. Here, we report synaptic plasticity deficits associated with cognitive inflexibility and CaMKIIα hyperactivity in Adnp-HT mice. These mice show impaired and inflexible contextual learning and memory, additional to social deficits, long after the juvenile-stage decrease of ADNP protein levels to ~10% of the newborn level. The adult Adnp-HT hippocampus shows hyperphosphorylated CaMKIIα and its substrates, including SynGAP1, and excessive long-term potentiation that is normalized by CaMKIIα inhibition. Therefore, *Adnp* haploinsufficiency in mice leads to cognitive inflexibility involving CaMKIIα hyperphosphorylation and excessive LTP in adults long after its marked expressional decrease in juveniles.

## Introduction

ADNP (activity dependent neuroprotective protein) is a transcription factor of the SWI/SNF remodeling complex [1]. ADNP is widely expressed in various tissues in rodents and humans [2] and regulates various biological processes, including embryogenesis [3], brain development [4], chromatin architecture [5], gene expression [6, 7], alternative splicing [8], Wnt/β-catenin signaling [9], autophagy [10], microtubule processes [11], axonal transport [12], and dendritic spines and actin filaments [13–16].

*ADNP* mutations have been associated with various neurodevelopmental abnormalities, including congenital anomalies, intellectual disability (ID), and autism spectrum disorders (ASD) [17]. Thus, it has been proposed that *ADNP*-related neurodevelopmental deficits be collected under the term ‘ADNP syndrome’ (https://rarediseases.info.nih.gov/diseases/12931/adnp-syndrome) (Helsmoortel-Van der Aa syndrome (HVDAS), MIM: 615873) [18, 19]. *ADNP* is one of the most frequently mutated ASD-risk genes [17, 19–22], accounting for ~0.17% of ASD cases [19]. *ADNP* is among the most penetrant ASD-risk genes, together with *CHD8*, *SCN2A*, *FOXP1*, and *SHANK3* (59–88%) [22].

Previous studies in *Adnp*-mutant mice have provided substantial insights into the pathophysiology underlying ADNP syndrome. Mice with homozygous deletion of exons 3–5 of *Adnp* (Adnp^∆3–5^ KO mice) display embryonic lethality [4], while Adnp^∆3–5^ heterozygous-mutant mice (Adnp^∆3–5^ HT mice) survive and exhibit learning and memory impairments, sociability deficits, social memory deficits, and social communication deficits [13, 23, 24]. An *Adnp*-mutant mouse line harboring the patient-derived p.Tyr719* mutation (Adnp^p.Tyr718*^ mice) displays sexually differential behavioral phenotypes [16]. These *Adnp* mouse phenotypes are rescued by the NAP peptide, which corresponds to eight aa residues of ADNP (NAPVSIPQ; also known as davunetide or CP201) [13, 16, 23] and is known to act through microtubule end-binding proteins [14].

Despite this substantial progress in our understanding, it remains largely unclear how ADNP deficiency leads to brain dysfunctions. For instance, it is unclear whether and how ADNP deficiency leads to impaired synaptic functions. A recent study has shown that juvenile-stage *Adnp* knockdown in mice alters prefrontal synaptic transmission [25], but whether embryonic-stage *Adnp* knockout in mice alters synaptic transmission and plasticity remains unclear. In addition, *Adnp* mRNA levels are decreased by ~four folds in the human brain during newborn and adult stages (https://hbatlas.org/) [26]. It is possible that similar changes may occur in the mouse brain and thus that *Adnp* gene deletion in mice may induce differential impacts at different postnatal stages (newborn, juvenile, and adult).

Here, we report that ADNP expression at newborn stages is markedly decreased (by ~90%) at juvenile and adult stages. We also compared the transcriptomic, proteomic, synaptic, and behavioral phenotypes at juvenile and adult stages in a new mouse line carrying a heterozygous deletion of exon 5 of *Adnp*. The results indicate that juvenile locomotor hyperactivity switches to adult hypoactivity, anxiety, and impaired and inflexible learning and memory. Regarding synaptic signaling, long-term depression (LTD) in juvenile mutants persisted into adulthood, whereas long-term potentiation (LTP) newly appeared in adults. These age-differential changes in synaptic plasticity accompanied hyperphosphorylation of CaMKIIα at the Thr286 autophosphorylation site. In addition, the enhanced LTP was normalized by CaMKIIα inhibition. These results suggest that *Adnp* haploinsufficiency in mice leads to CaMKIIα hyperactivity and cognitive inflexibility in adults.

## Results

### Behavioral abnormalities in newborn, juvenile, and adult *Adnp*-mutant mice

As a way to explore ADNP functions in vivo, we first determined the expression patterns of *Adnp* mRNAs and ADNP proteins in the mouse brain. *Adnp* mRNAs were detected in both glutamatergic and GABAergic neurons of various brain regions, including the cortex and hippocampus (Supplementary Fig. 1a). The ADNP protein, revealed by X-gal staining of the ADNP-β-galactosidase fusion protein in the *Adnp*-mutant mice (see below), was present in nearly all mouse brain regions, with relatively weak signals in the cerebellum (Supplementary Fig. 1b). During postnatal stages, ADNP protein levels were decreased to ~10% of the newborn level at the juvenile stage (~postnatal day (P) 21) (Supplementary Fig. 2a).

We next analyzed *Adnp*-mutant mice lacking a large portion of exon 5 containing the majority of the open-reading frame and functional domains of this protein (MGI:5050866; Adnp^tm1a(KOMP)Wtsi^) (Supplementary Fig. 2b–d). *Adnp*-heterozygous mutant mice (Adnp-HT mice) showed normal Mendelian ratios and ADNP protein expression of ~60% of that in WT mice; in this, they differed from *Adnp*-homozygous mice, which were embryonically lethal (Supplementary Fig. 2e).

In a battery of behavioral tests, Adnp-HT pups (~P7; males and females mixed) emitted fewer ultrasonic vocalizations (USVs) when separated from their mothers (Supplementary Fig. 3a**;** see Supplementary Table 1 for statistical details), indicative of early postnatal behavioral deficits. At juvenile stages (~3 weeks; males), Adnp-HT males showed hyperactivity in the open-field test but spent normal amounts of time in the center region of the open-field arena, which is a measure of anxiety-like behavior (Supplementary Fig. 3b, c). These mice showed decreased social interaction in the juvenile play test but normal levels of repetitive behaviors (self-grooming, digging, and jumping) (Supplementary Fig. 3d, e). Female Adnp-HT juveniles showed behavioral changes that are similar to those observed in males except for normal social interaction (Supplementary Fig. 3f–i).

Adult Adnp-HT mice (~2–6 months; males) showed hypoactivity in the open-field test (Supplementary Fig. 4a), contrary to the juvenile hyperactivity. These mice also showed anxiety-like behaviors in open-field (center time), elevated plus-maze, and light-dark tests (Supplementary Fig. 4b–d), and therein differed from juvenile Adnp-HT mice (normal open-field center time). The adult mice also showed social abnormalities, including decreased direct social interaction and abnormally increased courtship USV duration, although repetitive behaviors were largely normal (Supplementary Fig. 4e–g). In the Morris water maze test, adult Adnp-HT mice performed poorly in both the initial and reversal learning/probe phases (Supplementary Fig. 4h–j), which is in line with the frequent intellectual disability observed in ADNP syndrome [17]. The deficit was stronger in the reversal phase relative to the initial phase. Female adult Adnp-HT mice showed similar behavioral deficits, including hypoactivity, moderate anxiety-like behaviors, social-interaction deficits, and Morris water maze deficits, as compared with male mice (Supplementary Fig. 4k–s).

Therefore, the juvenile hyperactivity in Adnp-HT mice evolves to adult hypoactivity and anxiety-like behavior, whereas the social deficits are persistently observed. In addition, male and female Adnp-HT mice show impaired spatial learning and memory, with stronger phenotypes in the reversal phase, suggesting the presence of cognitive inflexibility. The largely similar behavioral deficits in adult mutant males and females differ from the sexual dimorphism previously reported in other lines of Adnp-HT mice (Adnp^∆3–5^ HT and Adnp^p.Tyr718*^ KI) [16, 24]. This is likely to reflect the distinct nature of the mutations. That said, a male:female ratio of 6:4 has been reported for ADNP-related ASD, which is milder than the 4.3:1 ratio seen in general ASD [17, 19].

### Altered neuronal excitability and synaptic transmission in juvenile and adult Adnp-HT hippocampal neurons

We hypothesized that the above-described behavioral changes observed in juvenile and adult Adnp-HT mice might involve altered neuronal excitability and/or synaptic functions. To address these possibilities, we first determined neuronal excitability in the hippocampus of juvenile and adult Adnp-HT mice.

Compared to wild-type, juvenile Adnp-HT mice (males) displayed decreased neuronal excitability in hippocampal CA1 pyramidal neurons, as shown by a current-firing curve, whereas there was no change in other parameters of neuronal excitability (input resistance and Sag ratio) (Supplementary Fig. 5a–d).

When synaptic transmissions were measured, compared to wild-type, juvenile Adnp-HT mice displayed increased frequency but not amplitude of miniature excitatory postsynaptic currents (mEPSCs) in CA1 pyramidal neurons (Supplementary Fig. 5e). In addition, there was a decrease in the amplitude but not frequency of miniature inhibitory postsynaptic currents (mIPSCs) (Supplementary Fig. 5f). Together, these changes increased the ratio of excitatory to inhibitory synaptic transmissions.

When spontaneous EPSCs (sEPSCs) and IPSCs (sIPSCs) were measured in the absence of tetrodotoxin to allow network activity, there was no genotype-related difference (Supplementary Fig. 5g, h), suggesting that network activity normalized the above-described differences.

Adult Adnp-HT mice displayed a similar decrease in CA1 neuronal excitability, again without any change in the input resistance or Sag ratio (Supplementary Fig. 5i–l). These results indicate that both juvenile and adult mutant mice exhibit decreases in neuronal excitability. Contrary to the results from juvenile Adnp-HT mice, however, adult Adnp-HT mice displayed no alteration of mEPSCs, mIPSCs, sEPSCs, or sIPSCs (Supplementary Fig. 5m–p).

These results collectively suggest that: (1) neuronal excitability is persistently decreased at juvenile and adult stages; (2) juvenile Adnp-HT mice show an increased ratio of excitatory to inhibitory synaptic transmission that is normalized by network activity; and (3) adult Adnp-HT mice do not show altered synaptic transmission at excitatory or inhibitory synapses.

### Altered synaptic plasticity in juvenile and adult Adnp-HT hippocampal neurons

**Fig. 1:**
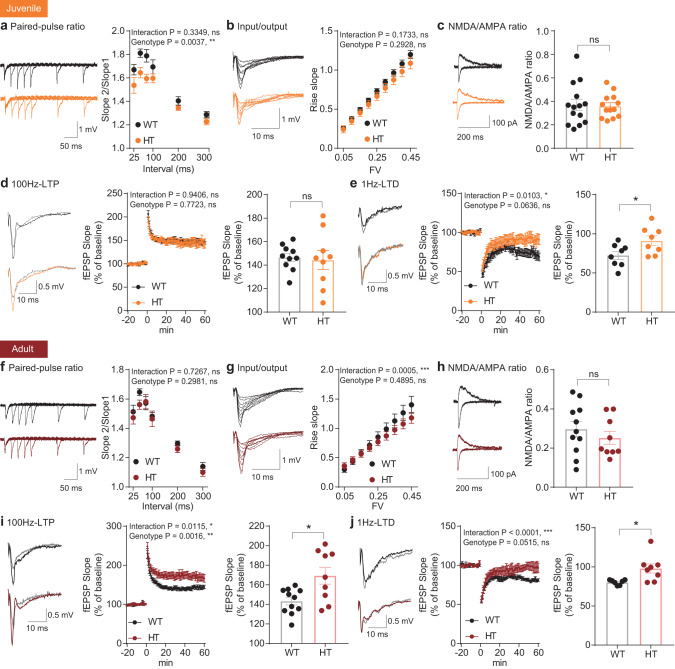
Altered synaptic plasticity in juvenile and adult Adnp-HT hippocampal neurons. **a** Decreased paired-pulse facilitation at hippocampal SC-CA1 synapses in juvenile Adnp-HT mice (~P21), as shown by slopes of fEPSPs (field excitatory postsynaptic potential) plotted against inter-stimulus intervals (*n* = 17 slices from 4 mice [WT], 17, 4 [HT], two-way RM-ANOVA). **b** Normal basal evoked excitatory synaptic transmission at juvenile Adnp-HT SC-CA1 synapses (~P21), as shown by an input-output curve plotting fEPSP amplitudes against fiber volley (FV) amplitudes (*n* = 14, 4 [WT], 12, 4 [HT], two-way RM-ANOVA). **c** Normal NMDA/AMPA ratios at juvenile Adnp-HT SC-CA1 synapses (~P21), shown by ratios of NMDAR- to AMPAR-mediated EPSCs (*n* = 14, 6 [WT], 13, 9 [HT], Student’s *t*-test). **d** Normal HFS-LTP at juvenile Adnp-HT SC-CA1 synapses (~P21). Gray, black, and orange colors in the voltage traces indicate baseline (in WT/HT), LTP (WT), and LTP (HT) synapses, respectively (*n* = 10, 5 [WT], 9, 6 [HT], two-way RM-ANOVA, Welch’s *t*-test (right, last 10 min)). **e** Suppressed LFS-LTD at juvenile Adnp-HT SC-CA1 synapses (~P21), (*n* = 8, 5 [WT], 8, 6 [HT], two-way RM-ANOVA with Bonferroni’s multiple comparison test, Student’s *t*-test [last 10 min]). **f** Normal paired-pulse facilitation at hippocampal SC-CA1 synapses in adult Adnp-HT mice (~4 months), as shown by fEPSP slopes plotted against inter-stimulus intervals (*n* = 11, 3 [WT], 9, 3 [HT], two-way RM-ANOVA). **g** Normal basal excitatory synaptic transmission at adult Adnp-HT SC-CA1 synapses (~4 months), indicated by the input-output curve of fEPSP amplitudes plotted against FV amplitudes. (*n* = 11, 3 [WT], 9, 3 [HT], two-way RM-ANOVA with Bonferroni’s multiple comparison test). **h** Normal NMDA/AMPA ratios at adult Adnp-HT SC-CA1 synapses (~2–3 months), shown by ratios of NMDAR- and AMPAR-EPSCs (*n* = 11, 7 [WT], 9, 6 [HT], Student’s *t*-test). **i** Enhanced HFS-LTP at adult Adnp-HT SC-CA1 synapses (3–4 months) (*n* = 11, 5 [WT], 9, 5 [HT], two-way RM-ANOVA with Bonferroni’s multiple comparison test, and Welch’s *t*-test (right, last 10 min)). **j** Suppressed LFS-LTD at adult Adnp-HT SC-CA1 synapses (3–4 months) (*n* = 7, 6 [WT], 8, 4 [HT], two-way RM-ANOVA, with Bonferroni’s multiple comparison test, and Student’s *t*-test [last 10 min]). Significance is indicated as *(<0.05), **(<0.01), ***(<0.001), or ns (not significant).

Given that Adnp-HT mice show suppressed learning and memory, we next tested if the Adnp-HT hippocampus displays alteration of evoked synaptic transmission and/or synaptic plasticity. Juvenile Adnp-HT mice displayed decreased paired-pulse facilitation at Schaffer collateral-CA1 (SC-CA1) synapses (Fig. 1a), suggestive of increased presynaptic release. In contrast, there was no genotype-related difference in evoked excitatory synaptic transmission, as shown by an input-output curve involving AMPA receptors (AMPARs), and postsynaptic receptor responses, as shown by the NMDA/AMPA ratio (ratio of NMDA receptor [NMDAR] to AMPAR-mediated EPSCs) (Fig. 1b, c).

When synaptic plasticity was measured, long-term potentiation induced by high-frequency stimulation (HFS-LTP) was not altered at SC-CA1 synapses in the juvenile Adnp-HT hippocampus (Fig. 1d). Intriguingly, however, long-term depression induced by low-frequency stimulation (LFS-LTD) was decreased at mutant SC-CA1 synapses (Fig. 1e). Therefore, juvenile Adnp-HT CA1 pyramidal neurons showed selectively increased presynaptic release and decreased LTD.

In adult Adnp-HT mice, SC-CA1 synapses displayed normal paired-pulse stimulation levels, input-output curves, and NMDA/AMPA ratios (Fig. 1f–h), suggesting that the increased presynaptic release in juvenile neurons was spontaneously normalized at adult stages. Intriguingly, the mutant SC-CA1 synapses displayed abnormally enhanced HFS-LTP (Fig. 1i), which was not observed in mutant juveniles, and suppressed LFS-LTD (Fig. 1j), which was observed in mutant juveniles.

These results collectively suggest that: (1) presynaptic deficits in juvenile Adnp-HT mice are normalized at adult stages; (2) the suppressed LTD in juvenile mutants persists into adulthood; and (3) enhanced LTP newly emerges in mutant adults.

### Synapse-related transcriptomic changes in Adnp-HT adults

To explore the mechanisms underlying juvenile and adult mutant mouse phenotypes in an unbiased manner, and considering that ADNP is a transcription factor, we next performed RNA-Seq analyses of the mutant hippocampus (Supplementary Table 2).

**Fig. 2:**
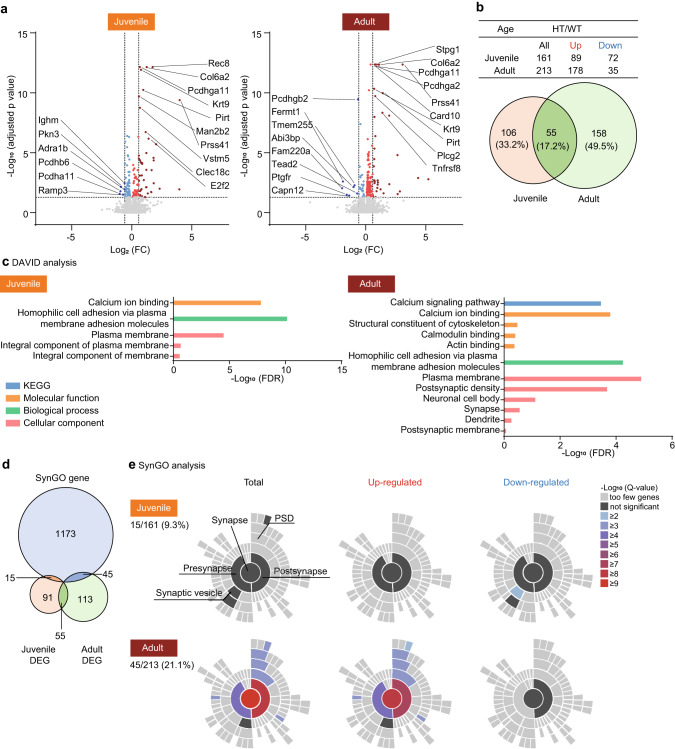
Synapse-related transcriptomic changes in the Adnp-HT juvenile and adult hippocampus as revealed by DEG analyses. **a** Volcano plots of DEGs (adjusted *p* < 0.05; light red/blue) in adult and juvenile Adnp-HT transcripts. Stroger DEGs with significant changes in *p* values and fold changes (FCs; threshold >1.5) are indicated in darker red/blue colors. **b** Numbers of up/downregulated DEGs in adult and juvenile Adnp-HT transcripts shown in the tables and Venn diagrams. **c** DAVID analyses of DEGs in adult and juvenile Adnp-HT transcripts, in domains of KEGG (Kyoto encyclopedia of genes and genomes), cellular component, biological process, and molecular function. **d** Venn diagrams showing the overlap between juvenile and adult Adnp-HT DEGs and SynGO genes. **e** SynGO analyses of DEGs in juvenile and adult Adnp-HT transcripts. Note that significant enrichment is observed only in upregulated adult DEGs, where postsynaptic enrichment is stronger than presynaptic enrichment.

The numbers of differentially expressed genes (DEGs; *p* < 0.05) were similar in juvenile and adult Adnp-HT transcriptomes (161 vs. 213) (Fig. 2a, b and Supplementary Table 3). The DEGs shared by the juvenile and adult Adnp-HT transcriptomes comprised ~30% of juvenile DEGs and ~25% of adult DEGs. While juvenile DEGs were composed of similar proportions of up- and downregulated DEGs (89 vs. 72), adult DEGs were more strongly upregulated (~ 5-fold; 178 vs. 35).

DAVID analyses of juvenile DEGs revealed enrichment for gene ontology (GO) functions associated with homophilic cell adhesion and calcium ion binding (Fig. 2c), suggestive of altered synaptic development and function at juvenile stages. Adult Adnp-HT DEGs were similarly enriched for GO functions such as homophilic cell adhesion, calcium ion binding, and calcium signaling, and, notably, for postsynaptic density and synapse, a term that was not detected in juvenile DAVID analysis.

Portions of both juvenile and adult Adnp-HT DEGs overlapped with SynGO (synaptic GO) genes [27] (Fig. 2d). However, significant SynGO enrichment was observed only in the upregulated adult DEGs but not in the downregulated adult DEGs or up/downregulated juvenile DEGs, where postsynaptic enrichment was stronger than presynaptic enrichment (Fig. 2e).

**Fig. 3:**
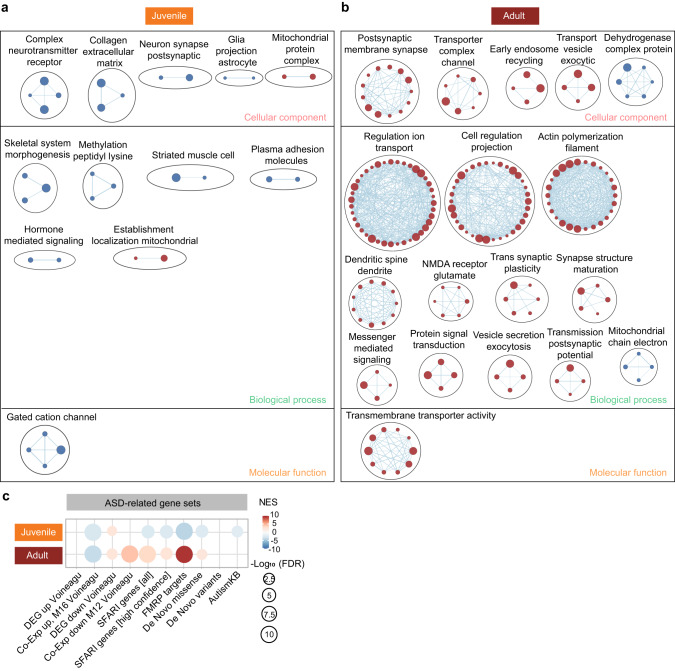
Synapse- and ASD-related transcriptomic changes in the Adnp-HT juvenile and adult hippocampus, as revealed by GSEA. GSEA results for juvenile (**a**) and adult (**b**) Adnp-HT transcripts using the gene sets in the cellular component (CC), biological process (BP), and molecular function (MF) domains of the C5 database (https://www.gsea-msigdb.org), as shown by enriched gene set clustering performed using Cytoscape EnrichmentMap App (https://cytoscape.org). The size of each node/circle indicates the number of genes in the gene set. The blue and red colors of the nodes indicate negative and positive enrichments, respectively. As the transcriptomic changes are stronger in adults than in juveniles, we show all the positive clusters for juveniles but only those with more than four gene sets for adults. **c** GSEA results for juvenile and adult Adnp-HT transcripts using ASD-related gene sets (DEG Up, Co-Exp Up M16, DEG Down, and Co-Exp Down M12), ASD-risk gene sets (SFARI, FMRP target, De Novo Missense, De Novo Variants, and AutismKB).

In gene set enrichment analysis (GSEA), which uses all transcripts (ranked by *p* value) to identify small but coordinated transcriptomic changes, juvenile Adnp-HT transcripts were moderately and negatively associated with gene sets associated with receptor protein complexes and extracellular matrix in the cellular component (CC) domain, as shown by clustering of enriched gene sets using Cytoscape EnrichmentApp (Fig. 3a, Supplementary Fig. 6, Supplementary Table 4). Juvenile Adnp-HT transcripts were also moderately and positively enriched for mitochondria- and ribosome-related gene sets.

Contrary to the juvenile patterns, GSEA of adult Adnp-HT transcripts indicated strong positive enrichments for synapse-related gene sets of the CC domain, such as the ‘neuron-to-neuron synapse’, ‘postsynaptic membrane’, and ‘glutamatergic synapse’ gene sets (Fig. 3b and Supplementary Fig. 6). Adult Adnp-HT transcripts were also negatively enriched for mitochondria/ribosome-related gene sets, which again contrasted with the juvenile patterns.

GSEAs of juvenile and adult Adnp-HT transcripts using the gene sets of the biological process (BP) and molecular function (MF) domains yielded largely dissimilar results, with synapse/ion transport-related gene upregulations being observed in adults (i.e., ‘regulation of synaptic plasticity’ and ‘trans-synaptic plasticity’ in the BP domain) (Fig. 3a, b and Supplementary Fig. 6). The strong positive enrichment of adult Adnp-HT transcripts for ‘regulation of ion transport’ function (BP) was mainly supported by calcium-related transmembrane and signaling proteins, including voltage-gated calcium channel subunit (*Cacna1h*), calcium release-activated calcium modulator 2 (*Orai2*), glutamate ionotropic receptor NMDA type subunit associated protein 1 (*Grina*), phosphoinositide interacting regulator of transient receptor potential channels (*Pirt*), ryanodine receptor 1 (*Ryr1*), phospholipase Cγ2 (*Plcg2*), neuron-specific calcium-binding protein hippocalcin (*Hpca*), and calcium-calmodulin kinase IIα (*Camkiia*).

These DEG and GSEA results collectively suggest that adult Adnp-HT transcripts are strongly associated with synapse-related functions.

### ASD-related transcriptomic changes in Adnp-HT juveniles and adults

Previous studies have identified many ASD-related/risk genes [28–30]. In GSEAs performed using these ASD-related/risk gene sets (Supplementary Table 5), juvenile Adnp-HT transcripts showed mixed changes that are consistent with and opposite to those occurring in ASD (termed ASD-like and reverse-ASD, respectively). The ASD-like changes found in mutant juveniles included negative enrichments for ASD-risk gene sets such as SFARI (all and high-confidence) [28], FMRP targets [31, 32], De Novo Missense (protein-disrupting or missense rare de novo variants) [31, 33], and AutismKB (Autism KnowledgeBase) [34, 35], which tend to be downregulated by various ASD-related mutations (Fig. 3c and Supplementary Table 4). However, there were some reverse-ASD changes, including negative enrichment of genes upregulated in ASD (Co-Exp Up M16) [31, 36] and positive enrichment of genes downregulated in ASD (DEG Down) [31, 36].

In adults, Adnp-HT transcripts showed strong reverse-ASD patterns, as shown by negative enrichment of genes upregulated in ASD (Co-Exp Up M16), positive enrichment of genes downregulated in ASD (DEG Down and Co-Exp Down M12), and positive enrichment of ASD-risk genes (SFARI, FMRP target, and De Novo Missense) (Fig. 3c). Of these changes, the positive enrichment for the FMRP-target gene set was particularly strong. The opposite enrichment patterns in juvenile and adult transcriptomes were mediated by both shared and distinct genes; i.e., ~50% of the genes in the FMRP target gene set showed opposite up/down regulations (Supplementary Fig. 6d, e).

These results collectively suggest that juvenile and adult transcriptomes show largely opposite patterns with respect to ASD-related transcriptomic changes, wherein the ASD-like patterns seen in juveniles change to reverse ASD-like patterns in adults.

### Synapse-related phospho-proteomic changes in the adult Adnp-HT hippocampus

**Fig. 4:**
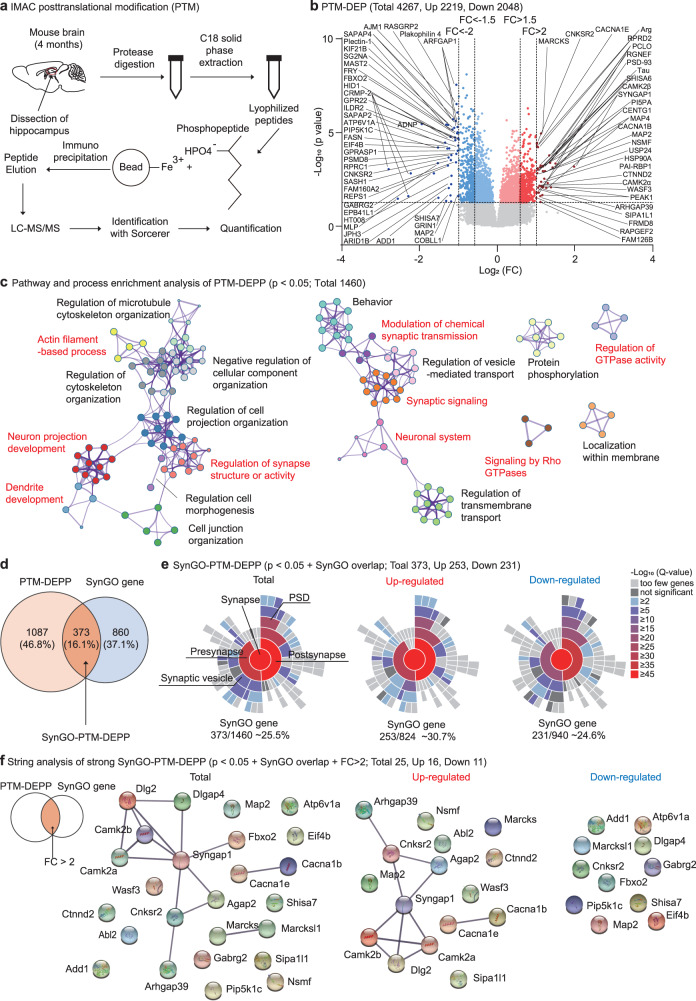
Synapse-related phospho-proteomic changes in the adult Adnp-HT hippocampus. **a** Schematic of phospho-proteomic analysis in adult male Adnp-HT and WT hippocampi (4 months). **b** Volcano plots of 4267 peptides (dots) with significant (*p* < 0.05) changes in PTM levels (PTM-DEPs; 2219 up and 2048 down) belonging to 1460 proteins (PTM-DEPPs; 824 up and 940 down) from adult Adnp-HT and WT mice (4 months). The PTM-DEPs with stronger changes (*p* < 0.05 + |fold change/FC| > 1.5 or 2.0) are indicated by darker red/blue colors, of which those that are |FC| > 2.0 are labeled of protein names. **c** Pathway and process analysis of PTM-DEPPs (*p* < 0.05; 1460 proteins) performed using the Metascape program (https://Metascape.org) and Cytoscape visualization. Synapse, actin, and small GTPase-related clusters are indicated in red. SynGO analysis indicates that 373 of the 1460 PTM-DEPPs belong to SynGO proteins (~26% of total, ~31% of upregulated, and ~25% of downregulated; termed SynGO-PTM-DEPPs) (**d**) and that they are localized at pre- and postsynaptic sites (**e**). **f** String analysis of SynGO-PTM-DEPPs (*p* < 0.05 and fold change >2.0; 25 total, 16 up and 11 down) for protein-protein interactions.

Given the stronger synapse-related transcriptomic changes observed in the adult Adnp-HT hippocampus, we next attempted a proteomic analysis of the adult Adnp-HT hippocampus. We first profiled posttranslational modifications (PTMs) (Fig. 4a and Supplementary Table 6), which are known to reflect various aspects of proteins, such as their synthesis, stability, localization, interaction, and degradation [37, 38].

Enrichment of phosphopeptides in Adnp-HT hippocampus using IMAC (immobilized metal ion affinity chromatography) yielded a total of 4267 peptides with significant (*p* < 0.05) changes in phospho-PTM levels (termed PTM-DEP [PTM-differentially expressed peptides]; 2219 up and 2048 down) belonging to 1460 proteins (824 up and 940 down) (Fig. 4b and Supplementary Table 7). Applying a stronger cutoff (*p* < 0.05 + fold change/FC > 2.0) yielded 30 upregulated and 39 downregulated PTM-DEPs (indicated in Fig. 4b by labeling).

DAVID analysis of the PTM-DEPPs (standing for PTM-DEP-containing protein; 1460 proteins; *p* < 0.05) revealed GO terms associated with neuronal synapses (cellular component), protein binding/phosphorylation, and intracellular signaling/GTPases/actin filaments (molecular function and biological process) (Supplementary Fig. 7).

Pathway and process analyses performed on the PTM-DEPPs using Metascape [39] highlighted synapse-related functions associated with neuronal synapses, actin filaments, and small GTPases (Fig. 4c); these functions were associated with both up- and downregulated DEPPs (Supplementary Fig. 8a, b). In addition, ‘regulation of transmembrane transport’ function was identified in the upregulated DEPPs, similar to the ‘regulation of ion transport’ function enriched in the RNA-Seq GSEA results (Fig. 3b).

A substantial fraction of the PTM-DEPPs (373 proteins from 1460 proteins; ~26%) belonged to the SynGO proteins (termed SynGO-PTM-DEPPs) (Fig. 4d); these proteins were similarly localized to pre- and postsynaptic compartments in the up- and downregulated groups (Fig. 4e). DAVID analysis of the SynGO-PTM-DEPPs (373 proteins) yielded synapse-related GO terms (Supplementary Fig. 9) stronger than those for PTM-DEPPs.

A string analysis of protein-protein interactions (PPIs) using the SynGO-PTM-DEPPs with a stronger cutoff (*p* < 0.05 + FC > 2.0; 25 proteins [16 up, 11 down] out of the total 373 proteins) revealed proteins known to assemble excitatory synaptic protein complexes, including an NMDAR subunit (*Grin2a*), scaffolding proteins (*Syngap1*, *Dlgap4*, *Cnksr2* [Rho-related scaffold], and *Map2*), and signaling molecules known to associate with or act downstream of NMDARs (*Camkiia* and *Camkiib*, *Agap2* [an Arf-GAP], and *Arhgap39* [a Rho GAP]) (Fig. 4f) [40].

These results collectively suggest that the adult Adnp-HT hippocampus displays phospho-proteomic changes that are associated with the regulation of excitatory synaptic structure and function.

### Comparison of total- and phospho-proteomic changes in the adult Adnp-HT hippocampus

To determine if the altered PTM levels reflect altered protein levels, altered PTM levels, or both, we attempted total (not PTM) proteome analyses in adult WT and Adnp-HT hippocampi (Supplementary Fig. 10a and Supplementary Table 8).

There were 449 differentially expressed proteins in the total Adnp-HT proteome (termed Total-DEPs; 270 upregulated, 179 downregulated) (Supplementary Fig. 10b and Supplementary Table 9). These numbers were relatively small relative to PTM-DEPPs (1460 proteins; 824 up and 940 down).

DAVID analysis of Total-DEPs (449 proteins) yielded GO terms related to synapse, protein binding, and GTPases/actin filaments (Supplementary Fig. 10c), partly similar to the results for PTM-DEPPs (Supplementary Figs. 7, 9). Pathway and process analyses of Total-DEPs (449 proteins) yielded small GTPase-related functions, although actin- and synapse-related functions were moderate and absent, respectively (Supplementary Fig. 10d).

Total-DEPs were enriched in SynGO proteins (69/449; ~15.4%; termed SynGO-Total-DEP), although to a lesser extent than PTM-DEPPs (373/1460; ~26% [~1.7-fold greater]) and only in downregulated Total-DEPs (Supplementary Fig. 10e, f). A string analysis for synaptic PPIs could not be performed because of the small number of SynGO-Total-DEPs (11 out of 69 SynGO-Total-DEPs when FC > 1.1 is used for cut-off).

In comparative analyses of Total-DEPs (*p* < 0.05; 449 proteins) and PTM-DEPPs (*p* < 0.05; 1460 proteins), a substantial fraction of Total-DEPs (114 proteins; ~25.4%) overlapped with PTM-DEPPs whereas a relatively small portion of Total-DEPs (~7.8%) overlapped with PTM-DEPPs (Supplementary Fig. 10g).

In a correlation plot, FCs in Total-DEPs (*p* < 0.05; 449 proteins) minimally correlated with those in PTM-DEPs (*p* < 0.05; 4267 peptides from 1460 proteins) (Supplementary Fig. 10h). Moreover, a substantial portion of the FCs (~27.5%) in PTM-DEPs occurred toward opposite directions compared with those in Total-DEPs (i.e., increased FCs in Total-DEPs but decreased FCs in their PTM-DEPs).

These results collectively suggest that Total-DEPs are less strongly associated with synaptic functions, compared with PTM-DEPPs, and that changes in PTM-DEPs are not strongly correlated with those in Total-DEPs and thus may contribute more strongly to the observed synaptic changes in the Adnp-HT hippocampus.

### CaMKII hyperphosphorylation in the Adnp-HT hippocampus

**Fig. 5:**
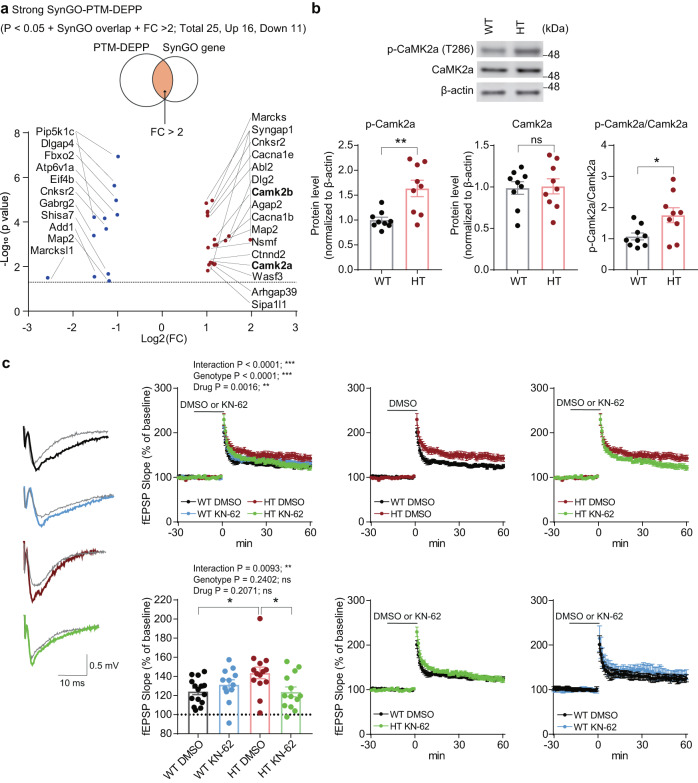
CaMKIIα hyperphosphorylation in the Adnp-HT hippocampus. **a** A volcano plot showing Adnp-HT SynGO-PTM-DEPPs (25 proteins found in both PTM-DEPs and SynGO proteins; *p* < 0.05 + FC > 2.0) plotted by their changes in PTM levels. The protein-encoding gene names are indicated. Note that both CaMKIIα and CaMKIIβ are hyperphosphorylated. **b** Validation of CaMKIIα hyperphosphorylation by immunoblot analysis. Adnp-HT lysates (crude synaptosomal/P2 fraction; 4 months) were immunoblotted for total CaMKIIα, Thr286-phosphorylated CaMKIIα (p-CaMKIIα), and β-actin (control) (*n* = 9 [WT], 9 [HT], Welch’s t-test for p-Camk2a and p-Camk2a/Camk2a, Student’s t-test for Camk2a). **c** Normalization of HFS-LTP at Adnp-HT Schaffer collateral (SC)-CA1 synapses (3–4 months) by the CaMKIIα inhibitor KN-62 (2.5 µM). Note that we lowered KN-62 concentration down to 2.5 µM and limited KN-62 treatment to 20 min before LTP induction to maximize the genotype difference in KN-62-dependent LTP inhibition. Gray, black, red, blue, and green colors in the voltage traces indicate baseline (in WT-DMSO/HT-DMSO/WT-KN-62/HT-KN-62), LTP (WT-DMSO), LTP (HT-DMSO), LTP (WT-KN-62) and LTP (HT-KN-62) synapses, respectively (*n* = 15 slices from 10 mice [WT-DMSO], 12, 9 [WT-KN-62], 14, 8 [Adnp-HT-DMSO], and 13, 10 [Adnp-HT-KN-62], three-way ANOVA for 60 min and two-way RM-ANOVA with Turkey’s multiple comparison test for last 10 min). Significance is indicated as *(<0.05), **(<0.01), ***(<0.001), or ns (not significant).

The abovementioned total- and phospho-proteomic changes in Adnp-HT mice indicate altered phosphorylations in synaptic proteins. We thus plotted the 25 SynGO-PTM-DEPPs in a volcano plot to see if we could identify notable changes in phosphorylation (Fig. 5a).

Most intriguingly, CaMKIIα showed increased phosphorylation at Thr286, an autophosphorylation site well-known to promote long-lasting enhancement of CaMKIIα activity and learning and memory [41–43]. An equivalent autophosphorylation in CaMKIIβ (Thr287) was also increased, although not indicated in the volcano plot or table for its FC < 2.0 (1.8). CaMKIIβ was also hyperphosphorylated at Thr321 and Ser331, although the former (Thr321) is known to promote cytosolic (not synaptic) localization [44].

Given the known importance of CaMKIIα Thr286 phosphorylation for synaptic regulation, we first validated the CaMKIIα hyperphosphorylation in Adnp-HT mice by immunoblot analysis. Thr286 phosphorylation levels of CaMKIIα were increased in the Adnp-HT hippocampus, without a change in the total CaMKIIα level, which increased the phospho/total-CaMKIIα ratio (Fig. 5b). The increase in Thr286 phosphorylation in the immunoblot analysis was smaller than the data obtained from the PTM analysis, likely reflecting the differences in the nature and sensitivity of the methods.

We next tested if the increased CaMKIIα Thr286 phosphorylation may contribute to the increased HFS-LTP observed at Adnp-HT hippocampal synapses using the CaMKIIα inhibitor KN-62. HFS-LTP at Adnp-HT synapses was increased in the absence of KN-62 (Adnp-HT-DMSO), as compared with WT synapses (WT-DMSO) (Fig. 5c), recapitulating the LTP results from naïve WT and Adnp-HT mice (Fig. 1i). Upon KN-62 treatment, HFS-LTP at Adnp-HT synapses (Adnp-HT-KN-62) was significantly reduced, compared with DMSO-treated Adnp-HT synapses (Adnp-HT-DMSO) (Fig. 5c). In contrast, KN-62 had no effect on WT LTP (WT-KN-62). These results suggest that the increased LTP at Adnp-HT synapses is attributable to increased CaMKIIα activity and that Adnp-HT synapses are more sensitive to KN-62 inhibition compared with WT synapses.

The CaMKIIα hyperactivity-dependent LTP enhancement at Adnp-HT synapses may involve CaMKIIα substrate proteins. When CaMKIIα substrate proteins in the literature were volcano plotted by their changes in phosphorylation levels (FC and *p* values) in the Adnp-HT PTM proteome, a total of 10 peptides in 7 proteins could be displayed (Supplementary Fig. 11a). These proteins/peptides tended to be upregulated than downregulated (5 vs. 2 in proteins; 8 vs. 2 in peptides), in line with the CaMKIIα hyperactivity. The downregulated phospho-peptides may suggest decreased total protein levels or co-phosphorylation by CaMKIIα and other kinases (i.e., Ryr2-Ser2807 phosphorylation [45]).

Of the upregulated proteins, two (SynGAP1 [*Syngap1*] and Synapsin 1 [*Syn1*]) displayed multi-site hyperphosphorylations (Supplementary Fig. 11a). One of the Synapsin 1 hyperphosphorylations could be validated by our immunoblot experiments where the phosphorylation level increased in Adnp-HT mice without changes in the total level (Supplementary Fig. 11b). Hyperphosphorylations were also found in other CaMKIIα substrate proteins known to regulate synaptic/neuronal functions, including voltage-gated calcium channel β2 (*Cacnb2*), metabotropic glutamate receptor 1 (*Grm1*), and nNOS (*Nos1*) [46–48].

The four hyperphosphorylated sites in SynGAP1 (Ser765, Ser766, Ser1111, and Ser1118) were mapped to the C-terminal DUF/Disordered domain (Supplementary Fig. 12a), known to modulate the SynGAP1 interaction with PSD-95 PDZ domains [49, 50]. These sites differed from the Ser1105/1135 residues, corresponding rat SynGAP1-Ser1108/1138, known to promote SynGAP1 dispersal from synapses during LTP [51]. However, our molecular modeling predicted that these phosphorylations can weaken the stability of the SynGAP1-PSD-95 complex (Supplementary Fig. 12b–e), suggesting that they may promote the dissociation of the SynGAP1-PSD-95 complex during LTP in Adnp-HT mice.

These results collectively suggest that the Adnp-HT proteome displays hyperphosphorylation of CaMKIIα, which may contribute to the synaptic alterations (i.e., enhanced LTP) in Adnp-HT mice.

## Discussion

Our study compared the molecular, synaptic, and behavioral phenotypes of juvenile and adult Adnp-HT mice lacking exon 5. Although there is a marked (~10-fold) decrease in Adnp expression at juvenile stages, the phenotypes of mutant mice continue to change during juvenile-to-adult stages, suggesting that *Adnp* haploinsufficiency at early developmental stages has long-lasting effects. The results also indicate abnormally enhanced LTP in adult Adnp-HT mice, which aligns with the impaired and inflexible spatial learning and memory. Our unbiased RNA-Seq and total/PTM proteomic analyses in adult ADNP-HT mice indicate hyperphosphorylations of CaMKIIα and its substrates (i.e., Synapsin 1). In addition, a moderate CaMKIIα inhibition normalizes LTP at mutant synapses without affecting LTP at WT synapses, suggesting that CaMKIIα hyperactivity enhances LTP and impairs learning and memory in Adnp-HT mice.

A key aspect of our study is the longitudinal comparisons of juvenile and adult Adnp-HT phenotypes at the molecular, synaptic, and behavioral levels. This approach revealed that the phenotypic changes continue to occur between the juvenile and adult stages (summarized in Supplementary Table 10). Age-differential ASD-like behaviors were observed in Adnp-HT juveniles (social interaction deficits and hyperactivity) and Adnp-HT adults (social interaction deficits, anxiety-like behavior, hypoactivity, and learning/memory deficits) (Supplementary Figs. 3, 4). These mice also display impaired and inflexible spatial learning and memory in the Morris water maze test, with stronger deficits in the reversal phase, which is in line with the cognitive inflexibility that lies at the core of ASD [52].

A key synaptic change identified in our study is the altered synaptic plasticity (both LTP and LTD) in the adult Adnp-HT hippocampus (Fig. 1). LTD is persistently decreased in both juvenile and adult Adnp-HT mice. The decreased LTD in juveniles may impair activity-dependent weakening or removal of less active excitatory synapses, likely leading to the observed increase in excitatory synaptic transmission and a decrease in neuronal excitability to balance the neuronal output (Supplementary Fig. 5). These changes may lead to the neuronal hyperconnectivity that has been implicated in ASD [53–55]. These changes may also explain the ASD-related social interaction deficits and behavioral hyperactivity seen in juvenile mutant mice, likely through the increased excitatory/inhibitory synaptic ratio implicated in ASD [56, 57].

LTP begins to be increased at adult (but not juvenile) stages (Fig. 1), which is likely to further suppress LTD considering that LTP is known to suppress LTD [58]. This dual impairment of synaptic plasticity (increased LTP and decreased LTD) in adult Adnp-HT mice is in good alignment with the impaired learning and memory observed in these mice. In previous studies, increased LTP has been shown to impair learning and memory [59, 60], and decreased LTD has been linked to impaired reversal learning and memory [61–63]. However, these results from the hippocampus should be considered together with those from other brain regions such as the prefrontal cortex [64] to fully understand the cognitive deficits in Adnp-HT mice.

Our results suggest molecular mechanisms that may underlie the increased LTP and decreased LTD in Adnp-HT mice. Altered NMDAR activity is an unlikely possibility because we observe normal NMDAR-mediated synaptic currents in Adnp-HT mice (Fig. 1). Alternatively, the signaling pathways acting downstream of NMDARs could be involved. In support of this possibility, our unbiased proteomic analyses (total and PTM) suggest a potential molecular candidate: hyperphosphorylation of CaMKIIα (Figs. 4, 5), which is a key component of excitatory synapses that regulates their assembly, transmission, plasticity, and signaling [41, 43, 65–67]. Specifically, our data indicate an increase in Thr286 phosphorylation (but not the total level) of CaMKIIα, which is known to be autophosphorylated and promote NMDAR-dependent synaptic potentiation [68, 69]. The CaMKIIα hyperphosphorylation would substantially change the phosphorylation profiles of downstream CaMKIIα substrates, thereby altering synaptic transmission and plasticity as well as learning and memory in the mutant mice. Indeed, LTP at Adnp-HT synapses is more sensitive to the CaMKIIα inhibitor KN-62, compared with that at WT synapses (Fig. 5c). In addition, mice carrying a phospho-mimicking mutation at Thr286 (Thr286Asp) has been shown to display impaired contextual discrimination [70].

Our PTM data reveal a number of potential CaMKIIα substrates that may contribute to the altered LTP/LTD seen in Adnp-HT mice. Related candidate proteins include SynGAP1; however, the phosphorylation sites altered in Adnp-HT mice did not match with previously described sites. For instance, there was no detectable phosphorylation of Ser1105/Ser1135 in SynGAP1, known to promote SynGAP1 dispersion out of the PSD during LTP [51, 71]. However, SynGAP1 in Adnp-HT mice display novel hyperphosphorylations at multiple sites (Supplementary Fig. 11a), which, by our molecular modeling, are predicted to weaken the SynGAP1 interaction with PSD-95 (Supplementary Fig. 12). Therefore, these phosphorylations may enhance SynGAP1 dispersal and disinhibition of the Ras signaling during LTP, given the reported association of SynGAP1 with LTP regulation and cognition [72–74].

It remains unclear how *Adnp* haploinsufficiency leads to CaMKIIα hyperphosphorylation. A possibility is that calcium signaling, lying upstream of CaMKIIα, was abnormally enhanced through various transcriptomic and proteomic changes induced by the insufficiency of the ADNP protein, which is a transcription factor. Specifically, calcium signaling is suggested by the results of 1) the RNA-Seq DAVID analysis (i.e., ‘calcium signaling’ and ‘calcium ion binding’) (Fig. 2c), 2) RNA-Seq GSEA analysis (‘regulation of ion transport’) (Fig. 3b), and 3) the PTM-DEPP Metascape analysis (‘regulation of transmembrane transport’) (Fig. 4c). This possibility is also supported by the enrichment of the Adnp-HT PTM-DEPs in the calcium signaling pathway (KEGG), which includes transmembrane calcium pump/channel proteins (PMCA/ATPase plasma membrane Ca2+ transporting 1 and CaV/voltage-gated calcium channel subunits α) and downstream calcium signaling pathway proteins (STIM/stromal interaction molecule 2, RYR/ryanodine receptor 2, CaMKs, and CaN/calcineurin) (Supplementary Fig. 13). Moreover, a recent study reported that ADNP regulates neuritogenesis through 14–3–3-dependent cytoplasmic localization and that in-utero *Adnp* knockdown leads to increased spontaneous calcium influx in female cortical neurons at P60 [75].

Although additional details remain to be investigated, our study is significant in the following four aspects: (1) We identify in Adnp-HT mice impaired and inflexible learning and memory, which lie at the core of the cognitive inflexibility associated with intellectual disability and ASD. (2) Although a recent study reported that acute *Adnp* knockdown induced synaptic changes in adult mice [25], we report for the first time (to our knowledge) that there are alterations in synaptic functions, particularly synaptic plasticity deficits, in juvenile and adult Adnp-HT mice. (3) We show that phenotypic changes occur between the juvenile and adult stages, long after the juvenile-stage marked decrease in ADNP expression, strengthening the notion that mutations in neurodevelopmental genes with strong early expression have long-lasting effects in adults after strong decreases of their expression [76]. (4) We provide a specific candidate mechanism, namely hyperphosphorylation of CaMKIIα, which may underlie the synaptic plasticity and behavioral deficits seen in Adnp-HT mice.

Clinically, our results from Adnp-HT mice may help us better understand the mechanisms underlying the cognitive and behavioral dysfunctions associated with ADNP-related developmental delay, ID, ASD, and ADHD [17]. In addition, the synaptic changes and accompanying CaMKIIα hyperactivity observed in Adnp-HT mice may eventually facilitate the development of mechanism-based ADNP treatments, given the known association of CaMKIIα dysfunction with various neurological and neuropsychiatric disorders and the ongoing development of related medications [77–80]. The synaptic changes observed herein might be associated with the davunetide (NAP) peptide that is known to improve Adnp-HT behavioral deficits in animal models for which the mechanistic details associated with potential synaptic targets remain unknown [81].

In summary, our results: (1) indicate that the molecular, synaptic, and behavioral phenotypes of Adnp-HT mice continue to develop from the juvenile to adult stages after a sharp decrease of *Adnp* expression; (2) associate altered synaptic plasticity with impaired and inflexible contextual learning and memory in Adnp-HT mice; and (3) suggest that hyperphosphorylation of CaMKIIα could underlie the synaptic and behavioral deficits in Adnp-HT mice.

## Methods and materials

### Animals

We purchased Adnp^Neomycin Cassette/+^ mice from KOMP (MGI:5050866; Adnp^tm1a(KOMP)Wtsi^) and backcrossed them with C57BL/6 J strains for more than five generations before we conducted our experiments. The generated mice were mated with Protamine-Flp mice, the resulting Adnp^fl/+^ mice were crossed with Protamine-Cre mice to generate mice with heterozygous deletion of Adnp exon 5 (Adnp-HT mice). We used Adnp-HT mice for the experiments because a homozygous deletion of exon 5 in the *Adnp* gene in mice caused lethality. Mice were bred and maintained at the mouse facility of Korea Advanced Institute of Science and Technology (KAIST) according to the Animal Research Requirements of KAIST, and all procedures were approved by the Committee of Animal Research at KAIST (KA2022-063-v1). Animals were fed ad libitum and housed under a 12-h light/dark cycle (light phase from 1:00 am to 1:00 pm). Genotypes of Adnp-HT mice were determined by polymerase chain reaction (PCR) using the following primer pairs: wild-type [WT] allele (413 bp), 5′-GAG AAA GAG AGC TGT TTG CCT TCC G-3′ (forward) and 5′-CAA GCA GTT ACA AGT TCC AGG CTC C-3′ (reverse); knock out (KO) allele (589 bp), 5-GAG AAA GAG AGC TGT TTG CCT TCC G-3′ (forward) and 5′-ATG CTG AGA AGG CAG GTA GGT AAG G-3′ (reverse); floxed allele (323 bp), 5′-GAG ATG GCG CAA CGC AAT TAA TG-3′ (forward) and 5′- CAA GCA GTT ACA AGT TCC AGG CTC C-3′ (reverse). Both male and female mice were used for behavior tests, but only male mice were used for other tests. Mice were weaned at the age of postnatal day 21, and mixed-genotype littermates in the same gender were housed together until experiments.

### X-gal staining

Coronal and sagittal sections of the mouse brain were prepared and sectioned by vibratome (Leica). Mice were perfused transcardially with 4% paraformaldehyde. The thickness of the brain slices was 250 μm. Slices were incubated in staining solution (5 mM K_3_Fe(CN)_6_, 5 mM K_4_Fe(CN)_6_•3H_2_O, 2 mM MgCl_2_, 0.01% deoxycholate, 1 mg/mL X-gal, 0.02% NP-40 in PBS) for 1 h 30 min at room temperature. Stained slices were washed four times with PBS and mounted for light microscopy.

### Fluorescent in situ hybridization (FISH)

FISH was performed as previously described [82]. Briefly, frozen mouse brain sections (14 µm thick) were cut coronally through the hippocampus and cortex formation. Sections were thaw mounted onto Superfrost Plus Microscope Slides (Fisher Scientific #12-550-15). The sections were fixed in 4% paraformaldehyde (PFA) for 10 min, dehydrated in increasing concentrations of ethanol for 5 min, and finally air-dried. Tissues were then pretreated for protease digestion for 10 min at room temperature. Probe hybridization and amplification were performed at 40 °C using HybEZ hybridization oven (Advanced Cell Diagnostics, Hayward, CA, USA). The probes used in these studies were five synthetic oligonucleotides complementary to the nt sequence 2–722 of Mm-Adnp-03-C1, 464–1415 of Mm-Slc17a7 (Vglut1)-C2,1986–2998 of Mm-Slc17a6 (Vglut2)-C3, 62–3113 of Mm-Gad1, and 552–1506 of Mm-Gad2 (Advanced Cell Diagnostics). The labeled probes were conjugated to Alexa Fluor 488, Atto 550, and Atto 647. The sections were hybridized with the labeled probe mixture at 40 °C for 2 h per slide. Unbound hybridization probes were removed by washing the sections three times with 1× wash buffer at room temperature for 2 min. The following steps for signal amplification included incubations at 40 °C with Amplifier 1-FL for 30 min, with Amplifier 2-FL for 15 min, with Amplifier 3-FL for 30 min and with Amplifier 4 Alt B-FL for 15 min. Each amplifier solution was removed by washing with 1× wash buffer at room temperature for 2 min. The slides were viewed, analyzed and photographed using TCS SP8 Dichroic/CS (Leica), and the ImageJ program (NIH) was used to analyze the images.

### Brain lysates and western blot

Mouse brains were extracted on ice and homogenized with ice-cold homogenization buffer (0.32 M sucrose, 10 mM HEPES, pH 7.4, 2 mM EDTA, pH 8.0, 2 mM EGTA, pH8.0, protease inhibitors, phosphatase inhibitors). Total lysates were prepared by boiling brain tissues with β-mercaptoethanol directly after homogenization. Crude synaptosomes, which were used for CaMK2 and Synapsin 1 immunoblotting, were prepared by centrifuging total lysates at 1500 × *g* followed by re-centrifuging the supernatant at 15,000 × *g* and retrieving the pellet (P2 fraction). Immunoblot conditions: ADNP (Bethyl; A300-104A; 1:1000), Camk2 (Cell signaling; 3362 S; 1:1000), p-Camk2 Thr286 (Abcam; 32678; 1:1000), Synapsin 1 (Thermo; 51-5200; 1:1000), pSynapsin 1 Ser605 (Cell Signaling; 88246 S; 1:1000), β-actin (Sigma; A5316; 1:2500), and PSD-95 (K28/43) (NeuroMAP; 75-028; 1:1000) at 4 °C overnight. Fluorescent secondary antibody signals were detected using Odyssey Fc Dual-Mode Imaging System.

### Behavior assays

All behavior assays were performed using littermates or sex- and age-matched animals, and conducted during light-off periods. Mice were allocated into specific cohorts at random, except genotype per cage post-weaning was set at a 1:1 ratio for WT vs. mutant. Male cohorts were caged separately from females when weaned. All experiments were done genotype blind-manner. Before the series of behavior assays were undertaken, adult mice were handled for 10 min per day for 3 days. Before each behavior assay, all mice were habituated in the dark room for 30 min. Different mouse cohorts were used for different sets of behavioral experiments, which were arranged in the order of increasing stress to animals (see Supplementary Table 11 for details).

### Open-field test

Mice were placed in the center of a white acrylic box (40 × 40 × 40 cm), and the activity of mice was recorded by a video camera for 1 h for adult behavior tests and 20 min for juvenile behavior tests. Illumination conditions for adult-male, adult-female, and juvenile tests were 100, 0, and 100 lux, respectively. The recorded video was analyzed using Ethovision XT13 software (Nodulus). The center zone was defined as an area of 30 × 30 cm in the whole area of 40 × 40 cm.

### Elevated plus-maze test

Mice were placed in the center zone of the elevated plus maze apparatus, and the activity of mice was recorded by a video camera for 8 min. The maze consisted of two closed arms (30 × 5 × 30 cm), two open arms (30 × 5 × 0.5 cm), and a center zone. The height of the maze was 75 cm from the floor, and the height of closed arm walls was 30 cm. The illumination of open arms was 200 lux. The recorded video was analyzed using Ethovision XT13 software (Nodulus).

### Light-Dark test

Mice were placed in the center of the light area of the light-dark test apparatus and the activity of mice was recorded by a video camera for 10 min. The apparatus was divided into light areas (21 × 29 × 20 cm) and dark areas (21 × 13 × 20 cm). Two areas were separated by an entrance (5 × 8 cm). The illumination of the light area was 600 lux. The recorded video was analyzed using Ethovision XT13 software (Nodulus).

### Repetitive behavior test

Mice were placed in the center of a fresh home cage with bedding and the activity of mice was recorded by a video camera for 20 min, and the last 10-min recordings were analyzed. The illumination was 60 lux. The repetitive behavior time was measured manually. We measured the time spent grooming, digging, and jumping.

### Direct social interaction test

Mice were isolated for 3 days in their home cage prior to direct social interaction test day. The direct social interaction test apparatus was a gray box (30 × 30 × 30 cm). Each mouse was habituated in the gray box for two consecutive days for 10 min. On day 3, genotype-matched pairs of mice were placed in each corner of a gray box. The social interaction time was measured manually. Total interaction included nose-to-nose interaction, nose-to-body interaction, following, body contact, allogrooming, and mounting.

### Juvenile play test

Mice were isolated for 30 min in a new home cage with bedding prior to the start of the test. Then, genotype- and sex-matched pairs of mice were placed in a new home cage without bedding. Mice were allowed to freely interact for 10 min. The social interaction time was measured manually. Total interaction included nose-to-nose interaction, nose-to-body interaction, following, body contact, allogrooming, and mounting.

### Ultrasonic vocalization (USV) test

Mice were isolated for 3 days in their home cage prior to USV test day. Each subject mouse’s home cage was placed into the USV chamber. Age-matched female mice (C57BL/6 J) were randomly introduced to each subject male mice’s cage. Subject male mice and intruder female mice freely interacted for 5 min. For the pup USV test, each pup (postnatal day 7) was isolated from the dam. The USV calls were recorded in the USV chamber for 3 min. Avisoft SASLab Pro software was used to analyze USVs. The number of USV calls and the mean duration of each call were analyzed. Signals were filtered from 1 Hz to 100 kHz and digitized with a sampling frequency of 250 kHz, 16 bits per sample (Avisoft UltraSoundGate 116H). To generate spectrograms, the following parameters were used (FFT length: 256, frame size: 100, window: FlatTop, overlap: 75%), resulting in a frequency resolution of 977 Hz and a temporal resolution of 0.256 msec. Frequencies lower than 25 kHz were filtered out to reduce background white noises [82].

### Morris water maze test

A hidden platform (10 cm diameter) was placed in a white plastic tank (120 cm diameter). Mice were trained to find the hidden platform 3 trials per day with an inter-trial interval of 30 min. The learning phase was performed on consecutive days until the latency to the platform is less than 20 s. The day next, we conducted a probe test (1 min) without the hidden platform. On the next day, we conducted reversal learning after changing the position of the platform to the opposite side. If latency to the platform is less than 20 s, the day next, we conducted a probe test (1 min) without a hidden platform. The recorded video was analyzed using Ethovision XT13 software (Nodulus). Time spent in quadrants and the number of platform passing were calculated.

### Brain slices for electrophysiology

Male mice at 3 weeks and at 2–4 months were anesthetized with isoflurane. Mouse brain sections (300 μm for whole cell patch clamp recording, 400 μm for extracellular field recording) were sectioned in ice-cold dissection buffer containing (in mM) 212 sucrose, 25 NaHCO_3_, 10 D-glucose, 2 Na-pyruvate, 1.25 ascorbic acid, 1.25 NaH_2_PO_4_, 5 KCl, 3.5 MgSO_4_, and 0.5 CaCl_2_ bubbled with 95% O2 and 5% CO2 gases using Leica VT 1200 vibratome. The slices were recovered for 30 min and maintained in artificial cerebrospinal fluid (ACSF) at 32 °C (in mM: 124 NaCl, 25 NaHCO_3_, 10 Glucose, 2.5 KCl, 1 NaH_2_PO_4_, 2.5 CaCl_2_, 1.3 MgSO_4_ oxygenated with 95% O2 and 5% CO2 gases). All recordings were performed after recovery for an additional 30 min at room temperature. During all recordings, brain slices were maintained in a submerge-type recording chamber perfused with 27.5–28.5 °C ACSF (2 ml min–1). Recording glass pipettes from borosilicate glass capillaries (Harvard Apparatus) were pulled using an electrode puller (Narishige).

### Whole-cell patch recordings

For whole-cell patch recordings in the hippocampal CA1 region, a recording pipette (2.5–3.5 MΩ) was filled with the internal solution (in mM: 100 CsMeSO_4_, 10 TEA-Cl, 8 NaCl, 10 HEPES, 5 QX-314-Cl, 2 Mg-ATP, 0.3 Na-GTP and 10 EGTA with pH 7.25, 295 mOsm for mEPSCs, sEPSCs and NMDA/AMPA ratio; 115 CsCl, 10 EGTA, 8 NaCl, 10 TEACl, 10 HEPES, 4 Mg-ATP, 0.3 Na-GTP, 5 QX-314 with pH 7.35, 295 mOsm for mIPSCs and sIPSCs; 137 K-gluconate, 5 KCl, 10 HEPES, 0.2 EGTA, 10 Na2-phosphocreatine, 4 Mg-ATP, 0.5 Na-GTP with pH 7.2, 280 mOsm for intrinsic excitability).

To measure mEPSCs, mIPSCs, sEPSCs, and sIPSCs, hippocampus CA1 pyramidal neurons were voltage-clamped at −70 mV. For mEPSCs and mIPSCs, picrotoxin (60 μM) and NBQX (10 μM) + APV (50 μM) were added to ACSF with TTX (1 μM), respectively. For sEPSCs and sIPSCs, picrotoxin (60 μM) and NBQX (10 μM) + APV (50 μM) without TTX were added, respectively. mE/IPSC and sE/IPSC events were selected based on the properties of the detected currents (rise time <1 ms, 10 pA <amplitude <500 pA, and decay half-width >2 ms). Responses were recorded for 2 min after maintaining a stable baseline for 5 min.

For neuronal excitability measurement, ACSF contained picrotoxin (60 μM), NBQX (10 μM), and AP5 (50 μM). First, minimal currents were introduced to hold the membrane potential around −70 mV in a current clamp mode. To evoke depolarizing voltage sag responses, increasing amounts of depolarizing step currents (by 10 pA, −150 to −10 pA) were injected. Then, to elicit action potentials, increasing amounts of depolarizing currents (0–330 pA) were injected in a stepwise manner. Input resistance was calculated as the linear slope of current-voltage plots generated from a series of increasing current injection steps.

For measuring NMDAR/AMPAR ratio, ACSF contained picrotoxin (60 μM). CA1 pyramidal neurons were voltage clamped at −70 mV, and EPSCs were evoked at every 15 s. AMPAR-mediated EPSCs were recorded at −70 mV, and 20 consecutive responses were recorded after a stable baseline. After recording AMPAR-mediated EPSCs, the holding potential was changed to +40 mV to record NMDAR-mediated EPSCs. The NMDA component was measured at 60 ms after the stimulation. The NMDA/AMPA ratio was determined by dividing the mean value of 15 NMDA components of EPSCs by the mean value of 20 AMPAR-mediated EPSC peak amplitudes.

All electric responses were amplified and filtered by Multiclamp 700B (Molecular Devices) and then digitized by Digidata 1550 (Molecular Devices). Data were acquired by Clampex 10.2 (Molecular Devices) and analyzed by Clampfit 10 (Molecular Devices).

### Extracellular field recordings

For extracellular field recordings in the hippocampal CA1 region, fEPSPs were recorded in the stratum radiatum of the hippocampal CA1 region using pipettes filled with ACSF. fEPSPs were amplified (MultiClamp 700B, Molecular Devices) and digitized (Digidata 1550, Molecular Devices) for measurements. Data were acquired by Clampex 10.2 (Molecular Devices) and analyzed by Clampfit 10 (Molecular Devices). The Schaffer collateral pathway was stimulated, and baseline responses were collected every 20 s with a stimulation intensity that yielded a half-maximal response. For input/output experiments, after acquiring a stable baseline, a series of increasing input stimuli were given to evoke output signals. Measured fEPSP slopes and fiber volleys were then interpolated by linear fits to plot input/output relationships. For paired-pulse ratio experiments, stimuli with indicated inter-pulse intervals (25, 50, 75, 100, 200, and 300 ms) were given, pairs of peak amplitudes were recorded, and the ratio of that amplitudes was calculated. To induce long-term potentiation (LTP) and long-term depression (LTD) at Schaffer collateral synapses on CA1 pyramidal neurons, high-frequency stimulation (100 Hz, 1 s) and low-frequency stimulation (1 Hz, 15 min) were applied, respectively. For LTP rescue experiments using the CaMKIIα inhibitor KN-62, hippocampal slices with 10-min baseline recordings were incubated with KN-62 (2.5 µM) or DMSO (control) in ACSF for 20 min, followed by HFS-LTP (100 Hz, 1 s) induction.

### RNA-Seq analysis

Mouse brains (3 weeks and 4 months) were isolated and dissected carefully on ice and immersed in RNAlater solution (Ambion, AM7020) to stabilize RNA. RNA extraction, library preparation, cluster generation, and sequencing were performed by Macrogen Inc. (Seoul, Korea). RNA samples were prepared for sequencing using a TruSeq RNA Sample Prep Kit v2 (Illumina) according to the manufacturer’s instructions. Sequencing was carried out using Illumina’s HiSeq 4000 to generate 101-bp paired-end reads. Image analysis and base calling were performed with the standard Illumina software RTA (Real-Time Analysis). The BCL (base calls) binary was converted into FASTQ utilizing Illumina package bcl2fastq.

Transcript abundance was estimated with Salmon (v1.1.0) (Patro et al., 2017) in a Quasi-mapping-based mode onto the Mus musculus genome (GRCm38) with GC bias correction (–gcBias). Quantified gene-level abundance data was imported to R (v.3.5.3) with the tximport [83] package and differential gene expression analysis was carried out using R/Bioconductor DEseq2 (v1.30.1) [84]. Normalized read counts were computed by dividing the raw read counts by size factors and fitted to a negative binomial distribution. The *p*-values were adjusted for multiple testing with the Benjamini–Hochberg correction. Genes with an adjusted *p*-value of less than 0.05 were considered differentially expressed. Volcano plots were generated using the R ggplot2 (v.3.1.1) package.

The Gene Ontology (GO) enrichment analyses were performed using DAVID software (version 6.8) [85]. Mouse gene names were converted to human homologs using the Mouse Genome Informatics (MGI) database (http://www.informatics.jax.org/homology.shtml). Gene Set Enrichment Analysis (GSEA) (http://software.broadinstitute.org/gsea) [86] was used to determine whether a priori-defined gene sets would show statistically significant differences in expression between WT and Adnp HT mice. Enrichment analysis was performed using GSEAPreranked® (gsea-3.0.jar) module on gene set collections downloaded from Molecular Signature Database (MSigDB v7.0). GSEAPreranked was applied using the list of all genes expressed, ranked by the fold change and multiplied by the inverse of the *p*-value with recommended default settings (1000 permutations and a classic scoring scheme). The false discovery rate (FDR) was estimated to control the false positive finding of a given normalized enrichment score (NES) by comparing the tails of the observed and null distributions derived from 1000 gene set permutations. The gene sets with an FDR of less than 0.05 were considered significantly enriched.

### IMAC analysis of phospho-proteome

Phospho-proteomic results for WT and Adnp-HT mouse brains were obtained using the IMAC (immobilized metal affinity chromatography) method (PTMScan Phospho-Enrichment IMAC Fe-NTA Magnetic Beads, Cell Signaling Technology).

Brain samples from three different mice were pooled to make n number of one. Briefly, mouse brain samples (4 months) containing the hippocampus were dissected on ice and snap-frozen in liquid nitrogen. Then brain samples were trypsin-digested and fractionated by solid-phase extraction. Digested phosphorylated peptides were enriched by IMAC and analyzed by LC-MS/MS [87, 88]. IMAC service uses positively charged ions, commonly Fe_3_^+^, to capture negatively charged phosphor-peptides. MS/MS spectra were evaluated using the SEQUEST and the Core platform from Harvard University. Assignment and relative quantification of the sequences to the MS/MS spectra was done with the Sorcerer program (Sage-N). Finally, the peptide sequence assignment was linked to parent ion peak intensities to measure approximate fold-changes in validated peptides between paired samples. DAVID Gene Ontology (GO) analysis was conducted using significantly changed motifs (*p* < 0.05) (http://david.ncifcrf.gov). For SynGO analysis, significantly changed motifs (*p* < 0.05) were extracted, and the gene names corresponding to the motifs (see Dataset EV1, ‘SynGO Input’ tab) were analyzed using the SynGO evidence-based resource for annotation of synaptic proteins (https://www.syngoportal.org/). *P*-values were adjusted for multiple comparisons using the false discovery rate approach. Data are visualized using sunburst graphs of localization (GO, cellular component). All raw MS data files have been deposited to the repository MassIVE (PXD037325).

### Total proteomic analysis

Trypsin-digested peptides from WT and Adnp HT brain samples (4 months) were labeled using 10-plex TMT reagent (Thermo Fisher Scientific, Rockford, IL) according to the manufacturer’s instructions. Then, samples were combined and fractionated into 20 fractions by basic reverse-phase liquid chromatography. For the liquid chromatography tandem mass spectrometry (LC-MS/MS) analysis, fractionated peptides were diluted by mobile phase A (99.9% water with 0.1% FA).

Dissolved samples were analyzed using an Orbitrap Exploris 480 mass spectrometer (Thermo Fisher Scientific) coupled to an UltiMate 3000 RSLCnano system (Thermo Fisher Scientific) equipped with a nano electrospray source. The mobile phases A and B were composed of 0 and 99.9% acetonitrile containing 0.1% formic acid, respectively. The LC gradient of 140 min at a flow rate of 250 nL/min was applied for the peptide separation. During the chromatographic separation, the Orbitrap Exploris 480 was operated in a data-dependent mode. The full scan resolution was 120,000 at m/z 400. The MS2 scans were performed with HCD fragmentation (37.5% collision energy). MS/MS spectra were analyzed using the following software analysis protocol with the Uniprot mouse database. The following parameters were used; a precursor mass tolerance of 5 ppm, a fragment ion mass tolerance of 600 ppm, two and more peptides assignments for protein identification at a false positive rate less than 0.01, static modification for the TMT 10-plex reagents (229.1629 Da) on the N-terminus and lysine residues, variable modification for oxidation (15.9949 Da) on methionine, and TMT reporter ion mass tolerance of 20 ppm. Statistical analysis was performed with Perseus software (version 1.6.15). The expressions of proteins between samples were compared using Welch’s *t*-test (*p* < 0.05). All raw MS data files have been deposited to the repository MassIVE (PXD036544).

### Structural analysis of the interaction between SynGAP1 and PSD-95

The peptide sequences relevant to serine residues putatively phosphorylated by CaMKII were selected from the phosphosite database (https://www.phosphosite.org/homeAction) [89]. The initial structural models for docking simulation were constructed using the crystal structure of PDZ1 (PDB ID 1KJW) and PDZ2 (PDB ID 5JXB) [90, 91] and PDZ3 (PDB ID 5JXB) [91] domains of the human PSD-95 protein by using PyMol software (Version 1.8) [92]. The peptides were then blind-docked into the target domains of PSD-95 (SH3 and PDZ3) using the HPEPDOCK web server [93]. The blind docking yielded ten complex structural models, which were then recalculated using RosettaDock-4.0 [94]. The natural and unphosphorylated peptide structures were utilized as an input model in the Vienna-PTM server (version 2.0) to generate the 3D structures of phosphorylated peptides [95]. The phosphorylated peptides were simulated, and energy minimization of phosphorylated peptides was performed using molecular dynamics (MD) with the GROMAC force field parameter, 54A8. The binding modes of the complex structures were then calculated under 25 °C temperature conditions using PRODIGY software to determine binding energies and affinities [96]. All of the depicted structures were generated using PyMol software (Version 1.8) [92].

### Statistical analysis

Sample sizes of each experiment were determined based on the relevant published literature and/or by the nature of the experimental design. Statistical analyses were performed using the GraphPad Prism 7 software. The normality of the data distribution was determined using the D’Agostino and Pearson omnibus normality test, and then the Student’s t-test (for a normal distribution and equal variance), Welch’s *t*-test (form a normal distribution and nonequal variance) or Mann–Whitney *U* test (for a non-normal distribution) was applied. If samples were dependent on each other, we used the paired t-test (for a normal distribution) or Wilcoxon signed-rank test (for a non-normal distribution). Equal variation or not were tested by *F*-test. Repeated measures of two-way ANOVA and subsequent Bonferroni post hoc multiple comparison tests, which were performed when there were significant interactions, were used for the time-varying analysis of results for the open-field test, Morris water maze test, input/output test, paired-pulse facilitation, LTP and LTD. Repeated measures three-way ANOVA and multiple comparison tests for the results with significant interactions were performed to analyze KN-62-dependent rescue results. Data with more than three groups were analyzed using one-way ANOVA. If a single value made the data distribution become non-normal and was detected as a significant outlier (*p < 0.05) by Grubb’s test, we removed the data as an outlier. The statistical significance of values is indicated in the figure panels as follows: *p < 0.05, **p < 0.01, ***p < 0.001. Detailed statistical results in Supplementary Table 1.

### Supplementary information

Supplementary Information

Supplementary Figure 1

Supplementary Figure 2

Supplementary Figure 3

Supplementary Figure 4

Supplementary Figure 5

Supplementary Figure 6

Supplementary Figure 7

Supplementary Figure 8

Supplementary Figure 9

Supplementary Figure 10

Supplementary Figure 11

Supplementary Figure 12

Supplementary Figure 13

Supplementary Table

## Data Availability

RNA-Seq results from Adnp-HT mice were deposited in the GEO (Gene expression Omnibus) database at NCBI under the accession number of GSE213354. Total proteomics data were deposited to the ProteomeXchange database under the accession number of PXD036544 (total) and PXD037325 (PTM).
