# Correction: *Adnp*-mutant mice with cognitive inflexibility, CaMKIIα hyperactivity, and synaptic plasticity deficits

**DOI:** 10.1038/s41380-023-02192-y

**Published:** 2023-07-21

**Authors:** Heejin Cho, Taesun Yoo, Heera Moon, Hyojin Kang, Yeji Yang, MinSoung Kang, Esther Yang, Dowoon Lee, Daehee Hwang, Hyun Kim, Doyoun Kim, Jin Young Kim, Eunjoon Kim

**Affiliations:** 1 Department of Biological Sciences, Korea Advanced Institute for Science and Technology (KAIST), Daejeon, 34141 Korea; 2 Center for Synaptic Brain Dysfunctions, Institute for Basic Science (IBS), Daejeon, 34141 Korea; 3 Division of National Supercomputing, Korea Institute of Science and Technology Information, Daejeon, 34141 Korea; 4 Research Center for Bioconvergence Analysis, Korea Basic Science Institute, 162 Yeongudanjiro, Ochang, Cheongju, Chungbuk 28119 Korea; 5 Therapeutics & Biotechnology Division, Drug discovery platform research center, Korea Research Institute of Chemical Technology (KRICT), Daejeon, 34114 Korea; 6 Department of Anatomy and BK21 Graduate Program, Biomedical Sciences, College of Medicine, Korea University, Seoul, 02841 Korea; 7 School of Biological Sciences, Seoul National University, Seoul, 08826 Korea; 8 Medicinal Chemistry and Pharmacology, Korea University of Science and Technology (UST), Daejeon, 34113 Korea

**Keywords:** Neuroscience, Molecular biology

Correction to: *Molecular Psychiatry* 10.1038/s41380-023-02129-5, published online 26 June 2023

Data availability

RNA-Seq results from Adnp-HT mice were deposited in the GEO (Gene expression Omnibus) database at NCBI under the accession number of GSE213354. Total proteomics data were deposited to the ProteomeXchange database under the accession number of PXD036544 (total) and PXD037325 (PTM).

The Data availability statement was incorrectly given as “RNA-Seq results from Adnp-HT mice were deposited in the GEO (Gene expression Omnibus) database at NCBI under the accession number of GSE213354 (reviewer token: mbcjwieophmdhit).” but should have been “RNA-Seq results from Adnp-HT mice were deposited in the GEO (Gene expression Omnibus) database at NCBI under the accession number of GSE213354.”

The original article has been corrected.

