# Supplementary material for: Adnp-mutant mice with cognitive inflexibility, CaMKIIα hyperactivity, and synaptic plasticity deficits
Source: Mol Psychiatry. 2023 Jun 26;28(8):3548–62. doi: 10.1038/s41380-023-02129-5 (PMC10618100; doi:10.1038/s41380-023-02129-5)
Supplement: Supplementary file 1 — Supplementary Information [file 41380_2023_2129_MOESM1_ESM.docx]

**Supplementary Information**

**Adnp-mutant mice with cognitive inflexibility, CaMKIIα hyperactivity, and synaptic plasticity deficits**

Heejin Cho ^1,2,#^, Taesun Yoo ^2,#^, Heera Moon ^1^, Hyojin Kang ^3^, Yeji Yang ^1,4^, MinSoung Kang ^5^, Esther Yang ^6^, Dowoon Lee ^7^, Daehee Hwang ^7^, Hyun Kim ^6^, Doyoun Kim ^5,8^, Jin Young Kim ^4^, and Eunjoon Kim ^1,2,*^

^1^Department of Biological Sciences, Korea Advanced Institute for Science and Technology (KAIST), Daejeon 34141, Korea; ^2^Center for Synaptic Brain Dysfunctions, Institute for Basic Science (IBS), Daejeon 34141, Korea; ^3^Division of National Supercomputing, Korea Institute of Science and Technology Information, Daejeon, 34141, Korea; ^4^Research Center for Bioconvergence Analysis, Korea Basic Science Institute, 162 Yeongudanjiro, Ochang, Cheongju, Chungbuk 28119, Korea; ^5^Therapeutics & Biotechnology Division, Drug discovery platform research center, Korea Research Institute of Chemical Technology (KRICT), Daejeon, 34114, Korea; ^6^Department of Anatomy and BK21 Graduate Program, Biomedical Sciences, College of Medicine, Korea University, Seoul 02841, Korea; ^7^School of Biological Sciences, Seoul National University, Seoul 08826, Korea; ^8^Medicinal Chemistry and Pharmacology, Korea University of Science and Technology (UST), Daejeon, 34113, Korea; ^#^These authors contributed equally to the study; *Corresponding author.

**Supplementary figure legends**

**Supplementary Figure 1.** **Expression patterns of Adnp mRNAs and ADNP proteins in the mouse brain.**

(a) Detection of Adnp mRNAs in Vglut1/2-positive glutamatergic neurons and Gad1/2-positive GABAergic neurons in the cortical and hippocampal regions of the mouse brain (2 months; male), revealed by double immunofluorescence in situ hybridization (FISH). Scale bar, 100 μm (left), 10 μm (middle), and 10 μm (right).

(b) Distribution patterns of ADNP proteins in the mouse brain (3 months; coronal and sagittal sections; male), as shown by X-gal staining. Scale bar, 1 mm.

**Supplementary Figure 2.** **ADNP protein expression patterns and** **generation and characterization of Adnp-HT mice.**

(a) Temporal expression patterns of ADNP proteins in whole brains of mice, as shown by immunoblot analysis. Note that ADNP protein levels are decreased to ~10% of newborn levels at ~P21 (juvenile stage), and this persists into adulthood. Data are shown as mean ± SEM. (n = 3 mice [WT] and 3 mice [HT], one-way ANOVA).

(b) Gene-targeting strategy for Adnp^Neomycin Cassette/+^ mice (from KOMP; MGI:5050866; Adnp^tm1a(KOMP)Wtsi^). A large portion of exon 5 in the *Adnp* gene was targeted for deletion. Note that the targeting construct is designed to fuse the Adnp protein with β-galactosidase (encoded by LacZ) to visualize Adnp protein expression by X-gal staining. Primers (forward/F and reverse/R) for PCR genotyping are indicated. Neo, neomycin resistance; LacZ, β-galactosidase; FRT, flippase recognition target; loxP, locus of x-over, P1.

(c) Exon 5 encodes the majority of the ADNP protein domains. NAP, octapeptide NAPVSIPQ; NLS, nuclear localization signal.

(d) PCR genotyping of WT and Adnp-HT mice (P56; male).

(e) ADNP protein levels are decreased to ~60% of WT levels in whole brains of Adnp-HT mice (2–3 months; male). (n = 3 mice [WT and HT], *p < 0.05, one-sample t-test).

**Supplementary Figure 3**. **Behavioral abnormalities in newborn and juvenile Adnp-HT mice.**

(a) Suppressed ultrasonic vocalizations (USVs) in Adnp-HT pups (males and females mixed; P7) separated from their mothers, as measured by the total call number and duration of each call. (n = 10 [WT], 7 [HT], Mann-Whitney U test (left), and Student’s t-test (right)).

(b) Hyperactivity in juvenile male Adnp-HT mice (3 weeks) in the open-field test. (n = 16 [WT], 15 [HT], two-way RM-ANOVA and Mann-Whitney U test).

(c) Normal anxiety-like behavior in juvenile male Adnp-HT mice (3 weeks), as measured by time spent in the center region of the open-field arena. (n = 16 [WT], 15 [HT], Student’s t-test).

(d) Decreased social interaction in juvenile male Adnp-HT mice (3 weeks) in the juvenile play test. (n = 9 [WT], 9 [HT], Student’s t-test).

(e) Normal repetitive behavior in juvenile male Adnp-HT mice (3 weeks), as shown by self-grooming, digging, and jumping in home cages with bedding. (n= 13 [WT], 12 [HT], Welch’s t-test for self-grooming, Student’s t-test for digging, and Mann-Whitney U test for jumping).

(f) Hyperactivity in juvenile female Adnp-HT mice (3 weeks) in the open-field test. (n = 25 [WT], 24 [HT], two-way RM-ANOVA and Student’s t-test).

(g) Normal anxiety-like behavior in juvenile female Adnp-HT mice (3 weeks), as measured by time spent in the center region of the open-field arena. (n = 25 [WT], 24 [HT], Student’s t-test).

(h) Normal social interaction in juvenile female Adnp-HT mice (3 weeks) in the juvenile play test. (n = 8 [WT], 8 [HT], Student’s t-test).

(i) Normal repetitive behavior in juvenile female Adnp-HT mice (3 weeks), as shown by self-grooming, digging, and jumping in home cages with bedding. (n= 9 [WT], 11 [HT], Student’s t-test for digging and Mann-Whitney U test for self-grooming and jumping).

Significance is indicated as * (< 0.05), ** (< 0.01), *** (< 0.001), or ns (not significant).

**Supplementary Figure 4. Behavioral abnormalities in adult Adnp-mutant mice.**

(a) Hypoactivity in adult Adnp-HT mice (male; 3-4 months) in the open-field test, as measured by the distance moved. (n = 14 [WT], 15 [HT], two-way RM-ANOVA and Student’s t-test).

(b) Anxiety-like behavior in adult Adnp-HT mice (male; 3–4 months), as measured by time spent in the open-field center. (n = 14 [WT], 15 [HT], Mann-Whitney U test).

(c) Anxiety-like behavior in adult Adnp-HT mice (male; 3–4 months), as measured by time spent in open arms of the elevated plus-maze test. (n = 25 [WT] and 21 [HT], Mann-Whitney U test (left), Wilcoxon matched-pairs signed rank test (right, WT), and Paired t-test (right, HT)).

(d) Anxiety-like behavior in adult Adnp-HT mice (male; 2–4 months) in the light/dark test, as measured by light-chamber time. (n = 34 [WT], 29 [HT], Student’s t-test).

(e) Decreased direct social interaction in adult Adnp-HT mice (male; 3–5 months), as measured by social interaction time. (n = 20 pairs [WT], 17 pairs [HT], Student’s t-test).

(f) Abnormally increased mean duration of USVs in adult Adnp-HT mice (male; 4–6 months) in the courtship USV test, as measured by the total call number and mean duration of each call. Note that only the mean duration of each call was increased. (n = 26 [WT], 20 [HT], Student’s t-test [total call number], Mann-Whitney U test [mean duration of calls]).

(g) Normal repetitive behavior in adult Adnp-HT mice (male; 3–5 months), as measured by time spent in self-grooming and digging in home cages with bedding. (n = 18 [WT], 15 [HT], Student’s t-test).

(h–j) Impaired spatial learning and memory in adult male Adnp-HT mice (4–5 months) in the acquisition/probe phases of initial and reversal learning/memory sessions in the Morris water maze (MWM) test, as shown by latency to platform (h), time spent in each quadrant (i,j), and number of platform crossings during the probe test (i,j). T, target; O, opposite; L, left; R, right; R1/2/3, reversal day 1/2/3. (n = 15 [WT], 14 [HT], two-way RM-ANOVA with Bonferroni's multiple comparison test (h), Friedman test and Dunn’s multiple comparison test (i; forward learning [WT], [HT], j; reversal learning [HT]), one-way RM-ANOVA (j; reversal learning [WT]), and Student’s t-test (i; platform crossing), and Welch’s t-test (j; platform crossing).

(k) Hypoactivity in female adult Adnp-HT mice (3–4 months) in the open-field test, as measured by distance moved. (n = 15 [WT], 13 [HT], two-way RM-ANOVA with Bonferroni's multiple comparison test and Student’s t-test).

(l) Normal anxiety-like behavior in female adult Adnp-HT mice (3–4 months), as measured by open-field center-zone time. (n = 15 [WT], 13 [HT], Mann-Whitney U test).

(m) Normal anxiety-like behavior in female adult Adnp-HT mice (3–5 months), as measured by open-arm time in the elevated plus maze test. (n = 16 [WT] and 12 [HT], Student’s t-test (left), and paired t-test (right)).

(n) Anxiety-like behavior in female adult Adnp-HT mice (3–5 months) in the light/dark test, as measured by light-chamber time. (n = 17 [WT], 12 [HT], Student’s t-test).

(o) Decreased direct social interaction in female adult Adnp-HT mice (3–5 months), as measured by social interaction time. (n = 8 pairs [WT], 6 pairs [HT], Welch's t-test).

(p) Normal repetitive behavior in female adult Adnp-HT mice (3 months), as measured by self-grooming and digging times in home cages with bedding. (n = 8 [WT], 6 [HT], Student’s t-test (self-grooming), and Welch’s t-test (digging)).

(q–s) Impaired spatial learning and memory in adult female Adnp-HT mice (2–4 months) in the acquisition/probe phases of initial and reversal learning/memory sessions of the MWM test, as shown by latency to platform (q), time spent in each quadrant (r,s), and number of platform crossings (r,s). (n = 18 [WT], 19 [HT], two-way RM-ANOVA (q), one-way RM-ANOVA (r; forward learning [HT], s; reversal learning [WT], [HT]), Friedman test and Dunn’s multiple comparison test (r; forward learning [WT]), and Student’s t-test (r and s; platform crossing).

Significance is indicated as * (< 0.05), ** (< 0.01), *** (< 0.001), or ns (not significant).

**Supplementary Figure 5.** **Altered neuronal excitability and synaptic transmission in juvenile and adult Adnp-HT hippocampal neurons.**

(a–d) Decreased neuronal intrinsic excitability in hippocampal CA1 pyramidal neurons in juvenile Adnp-HT mice (P21; male), as shown by current-firing curve (d), but normal input resistance (a and b) and sag ratio (c). (n = 14 neurons from 3 mice [WT], 18, 3 [HT], two-way RM-ANOVA with Bonferroni's test (for (a) and (d)), Mann-Whitney U test (for (b)) and Student’s t-test (for (c)).

(e) Increased frequency but normal amplitude of mEPSCs in CA1 pyramidal neurons in juvenile Adnp-HT mice (P21). (n = 15 neurons from 3 mice [WT], 15, 3 [HT], Welch's t-test (frequency), and Student’s t-test (amplitude)).

(f) Decreased amplitude but normal frequency of mIPSCs in juvenile Adnp-HT CA1 pyramidal neurons (P21). (n = 22, 4 [WT], 24, 4 [HT], Student’s t-test).

(g) Normal frequency and amplitude of sEPSCs in juvenile Adnp-HT CA1 pyramidal neurons (P21). (n = 13, 3 [WT], 15, 3 [HT], Student’s t-test).

(h) Normal frequency and amplitude of sIPSCs in juvenile Adnp-HT CA1 pyramidal neurons (P21). (n = 14, 3 [WT], 20, 3 [HT], Mann-Whitney U test (frequency), Student’s t-test (amplitude)).

(i–l) Decreased neuronal intrinsic excitability in hippocampal CA1 pyramidal neurons in adult Adnp-HT mice (3–4 months; male), as shown by current-firing curve (l), but normal input resistance (i and j) and sag ratio (k). (n = 15, 4 [WT], 17, 4 [HT], two-way RM-ANOVA (for (i) and (l)), Bonferroni's test (for (l)), Student’s t-test (for (k)), and Welch’s t-test (for (j))).

(m) Normal frequency and amplitude of mEPSCs in adult Adnp-HT CA1 pyramidal neurons (3–4 months). (n = 12, 5 [WT], 14, 4 [HT], Student’s t-test).

(n) Normal frequency and amplitude of mIPSCs in adult Adnp-HT CA1 pyramidal neurons (3–4 months). (n = 12, 4 [WT], 14, 3 [HT], Student’s t-test).

(o) Normal frequency and amplitude of sEPSCs in adult Adnp-HT CA1 pyramidal neurons (2–3 months). (n = 15, 4 [WT], 16, 4 [HT], Student’s t-test).

(p) Normal frequency and amplitude of sIPSCs in adult Adnp-HT CA1 pyramidal neurons (2–3 months). (n = 13, 3 [WT], 12, 3 [HT], Student’s t-test (for frequency), and Welch’s t-test (for amplitude)).

Significance is indicated as * (< 0.05), ** (< 0.01), *** (< 0.001), or ns (not significant).

**Supplementary Figure 6. GSEA results for biological functions in the juvenile and adult Adnp-HT hippocampus.**

(a–c) Results of GSEA for juvenile (a) and adult (b and c) Adnp-HT transcripts using the gene sets in the cellular components (CC), biological process (BP), and molecular function (MF) domains of the C5 database (https://www.gsea-msigdb.org), as shown by the top-five most strongly enriched gene sets (a and b) and Cytoscape EnrichmentMap App (<https://cytoscape.org>) (the juvenile Cytoscape results are omitted because they are identical to those shown in main **Fig. 3a**).

(d and e) Up and downregulations of genes in the FMRP target gene set in juvenile and adult Adnp-HT transcripts indicated by bar graphs and fold-change distributions.

**Supplementary Figure 7.** **DAVID analysis of Adnp-HT PTM-DEPPs.**

(a) DAVID analysis of PTM-DEPPs (1460 proteins) for KEGG pathways and the GO terms, cellular component (CC), biological process (BP), and molecular function (MF).

**Supplementary Figure 8.** **Pathway/process analyses of up/downregulated Adnp-HT PTM-DEPPs.**

(a and b) Pathway and process analyses of up- and downregulated PTM-DEPPs (p < 0.05; 824 upregulated and 940 downregulated proteins) were performed using the Metascape program and Cytoscape visualization. Synapse, actin, and small GTPase-related clusters are indicated in red.

**Supplementary Figure 9.** **DAVID analysis of SynGO-PTM-DEPPs.**

(a) DAVID analysis of SynGO-PTM-DEPPs (PTM-DEPPs that belong to SynGO proteins; 373 proteins) for KEGG pathways and the GO terms cellular component (CC), biological process (BP), and molecular function (MF).

**Supplementary Figure 10. Total proteomic changes and comparison with phosphor-proteomic changes in the adult Adnp-HT hippocampus**.

(a) Schematic depicting our analysis of total protein patterns in the hippocampal regions of adult Adnp-HT and WT mice (4 months; male). TMT, Tandem Mass Tag; IP2, Integrated Proteomics Pipeline.

(b) Volcano plots of all proteins showing differential expression (termed Total-DEPs; p < 0.05; 449 total, 270 upregulated, and 179 downregulated) from adult Adnp-HT and WT mice (4 months). Total-DEPs with stronger changes (p < 0.05 and fold change [FC] > 1.2 and 1.5) are indicated by darker red/blue colors.

(c) DAVID analysis of Adnp-HT Total-DEPs (p < 0.05; 449 total, 270 upregulated, and 179 downregulated proteins) for GO terms associated with KEGG pathways and cellular components (CCs), biological processes (BPs), and molecular function (MFs).

(d) Pathway and process analysis of the total Total-DEPs (p < 0.05; 449 proteins [270 up and 179 down]) performed using the Metascape program and Cytoscape visualization. Synapse, actin, and small GTPase-related clusters are indicated in red.

(e and f) SynGO analysis indicates that 69 of the 449 Total-DEPs (p < 0.05) belong to SynGO proteins (~15.4%; termed SynGO-Total-DEP) and that these proteins are localized more strongly at postsynaptic sites than at presynaptic sites.

(g) A Venn diagram showing the triple overlaps among PTM-DEPPs (p < 0.05; 1460 proteins), Total-DEPs (p < 0.05; 449 proteins), and SynGO proteins (1233 proteins).

(h) Two-dimensional comparison of the FCs in PTM-DEPs (p < 0.05; 4267 peptides from 1460 proteins) and Total-DEPs (p < 0.05; 449 proteins). Note that there are limited correlations between PTM-DEPs and Total-DEPs in all quadrants. Note also that: 1) PTM-DEPs display greater FCs than Total-DEPs, as shown by the correlation coefficients for each of the four quadrants (Pearson’s test); and 2) 221 PTM-DEPs (~72.5%; from 89 proteins) change in the same direction between the genotypes (either increase/decrease), whereas 84 PTM-DEPs (~27.5%; from 50 proteins) change in opposite directions.

**Supplementary Figure 11. Known substrates of CaMKIIα in Adnp-HT PTM-DEPs and validation of synapsin 1 Ser605 phosphorylation.**

(a) A volcano plot showing Adnp-HT PTM-DEPs that overlap with known (not predicted) CaMKII substrate proteins and are significant (p < 0.05) in their changes in PTM levels.

(b) Validation of synapsin 1 hyperphosphorylation at Ser605 by immunoblot analysis. Adnp-HT lysates (crude synaptosomal/P2 fraction; 4 months) were immunoblotted for total synapsin 1, Ser605-phosphorylated synapsin 1 (p-Synapsin1), and β-actin (control). (n = 9 [WT and HT]; Mann-Whitney U test for p-synapsin 1, Student’s t-test for synapsin 1, Welch’s t-test for p-synapsin 1/synapsin 1).

**Supplementary Figure 12. Structural docking simulation for the interactions between SynGAP1 phospho-peptides and PSD-95 PDZ domains.**

(a) The domain structure (PH, C2, RasGAP, and DUF) of the mouse SynGAP1 protein (NP_001268420.1) with known/predicted CaMKII p-Ser sites. The phospho-Ser sites in Adnp-HT mice with significant increases (P < 0.05; FC > 1.4) and insignificant changes are indicated by magenta and black colors, respectively.

(b) A table summarizing the free energy (ΔG in kcal/mol; a measure of structural stability) and affinity of the complexes between the indicated SynGAP1 peptides (unphosphorylated-Ser [Nat], Ser-to-Asp mutation mimicking phosphorylated states [SD], and phosphorylated Ser [pS]) and the PDZ1/2/3 domains of PSD-95. Blue and red colors represent relatively strong and weak stability of the SynGAP1-PSD-95 complexes, respectively. Phospho-target serine residues in the peptide sequences are highlighted in red.

(c–e) Modeled structures of the complexes between SynGAP1 peptides (Nat, SD, and pS at pS765, pS766, pS1111, and pS1118 residues) and the PDZ1, PDZ2, and PDZ3 domains of PSD-95. The Nat, SD, and pS residues are indicated in yellow, blue, and red, respectively, where pS areas are highlighted by a yellow circle.

**Supplementary Figure 13. Enrichment of Adnp-HT PTM-DEPs in the calcium signaling pathway.**

(a) Enrichment of Adnp-HT PTM-DEPs in the calcium signaling pathway (KEGG). Significantly (p < 0.05) altered phosphorylations of calcium signaling pathway proteins are indicated in the boxes (red/blue/violet for increased/decrease/mixed), which include transmembrane calcium pump (i.e., PMCA [*Atp2b1/2*])/channel (i.e., CaV1/2/voltage-gated calcium channel α and β subunits [*Cacna1a/b/c/e* and *Cacnb1/2/4*]) proteins and downstream cytoplasmic calcium signaling pathway proteins (i.e., STIM [*Stim2*], RYR [*Ryr2*], CAMK [*Camk2a/b/d/g*], and CaN/calcineurin [*Ppp3ca*]). Note that the Thr498 phosphorylation in voltage-gated calcium channel subunit *β*2 [Cacn2b], known to active channel function, can be phosphorylated by CaMKIIα, suggestive of a positive feedback loop.

**Supplementary tables**

**Supplementary Table 1. Statistical details.**

**Supplementary Table 2. Raw RNA-Seq data from the hippocampal region of juvenile and adult Adnp-HT mice.**

**Supplementary Table 3. DEGs from the hippocampal transcripts in juvenile and adult Adnp-HT mice.**

**Supplementary Table 4. GSEA results for the hippocampal transcripts in juvenile and adult Adnp-HT mice.**

**Supplementary Table 5. List of ASD-related/risk gene sets used for GSEA.**

**Supplementary Table 6. Raw PTM data from WT and Adnp-HT mice.**

**Supplementary Table 7.** **Peptides with differentially expressed PTMs in WT and Adnp-HT mice (PTM-DEPs).**

**Supplementary Table 8. Raw total proteomics data from WT and Adnp-HT mice.**

**Supplementary Table 9. Total proteins differentially expressed in WT and Adnp-HT mice (Total-DEPs).**

**Supplementary Table 10. Summary of the behavioral and electrophysiological results from the current study.**

**Supplementary Table 11. The order of behavior experiments performed.**
