# Supplementary figures and images for: Adnp-mutant mice with cognitive inflexibility, CaMKIIα hyperactivity, and synaptic plasticity deficits
Source: Mol Psychiatry. 2023 Jun 26;28(8):3548–62. doi: 10.1038/s41380-023-02129-5 (PMC10618100; doi:10.1038/s41380-023-02129-5)

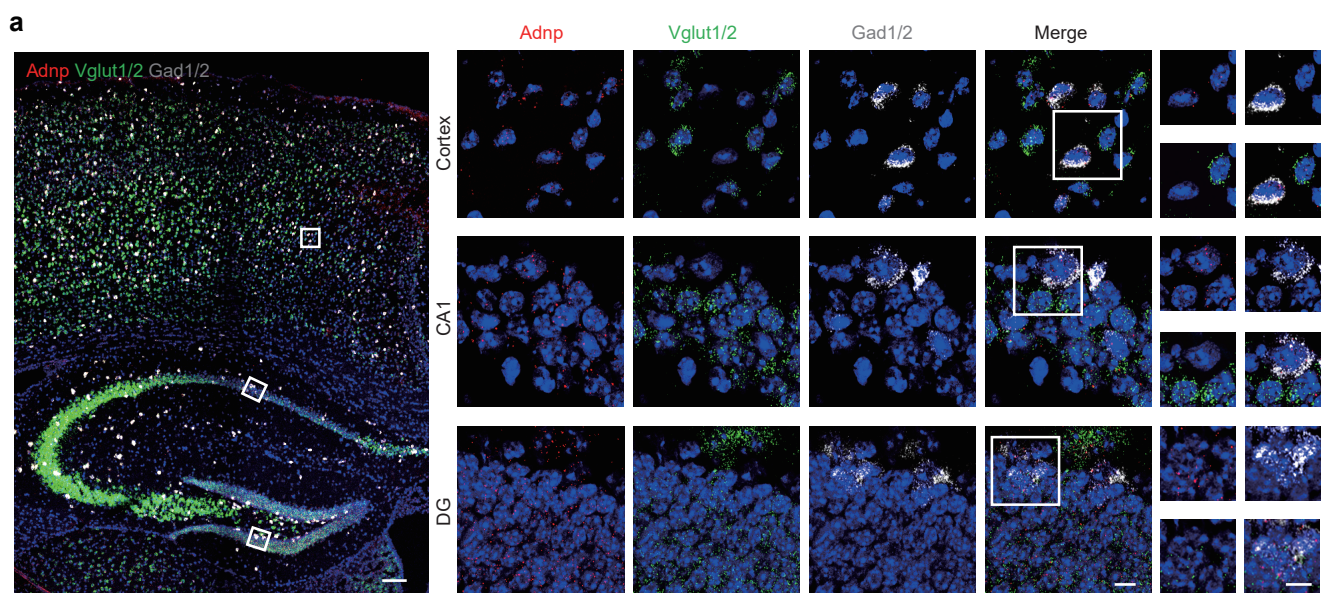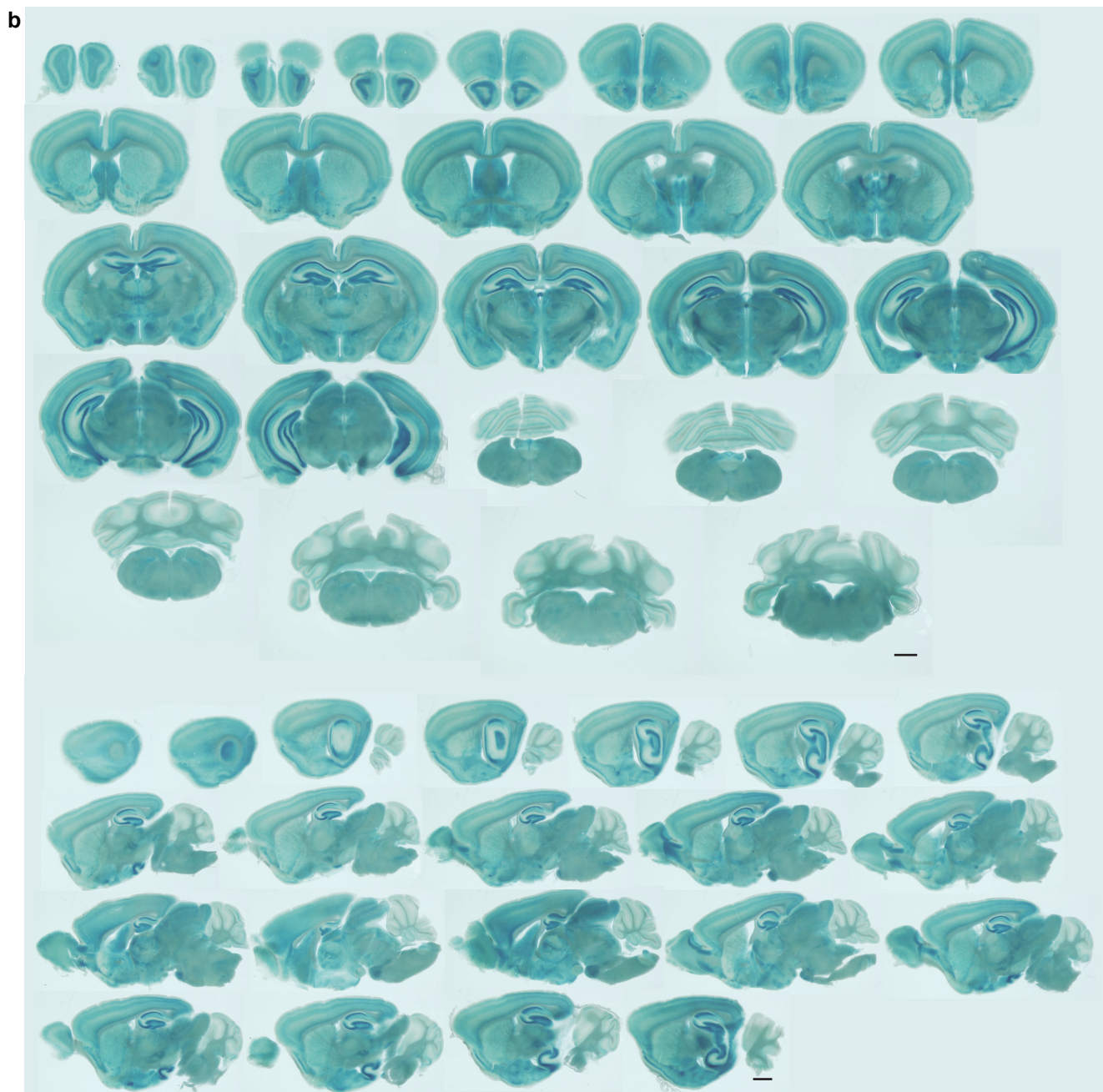

Supplement: Supplementary file 2 — Supplementary Figure 1 [file 41380_2023_2129_MOESM2_ESM.pdf]

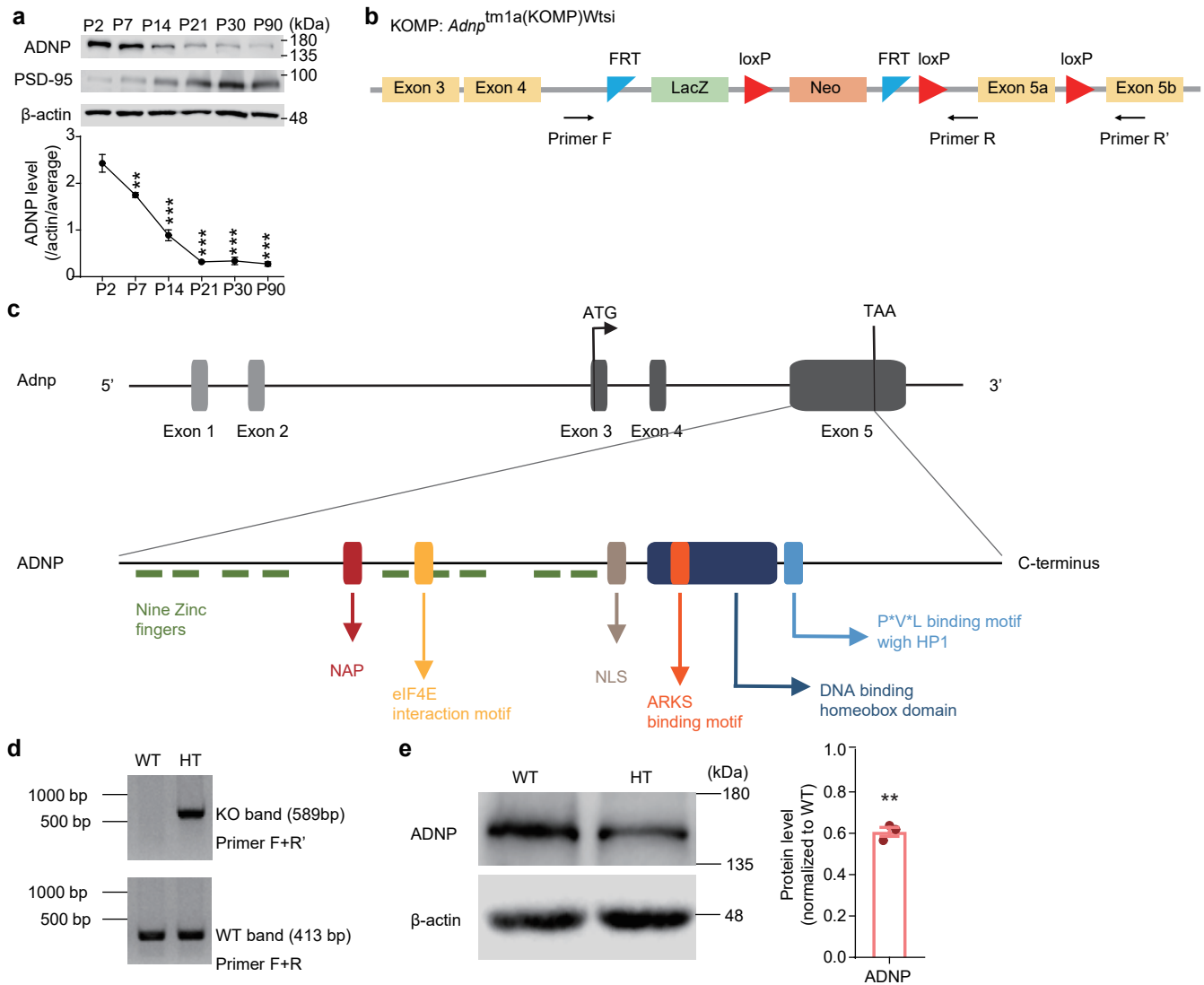

Supplement: Supplementary file 3 — Supplementary Figure 2 [file 41380_2023_2129_MOESM3_ESM.pdf]

# Adult male

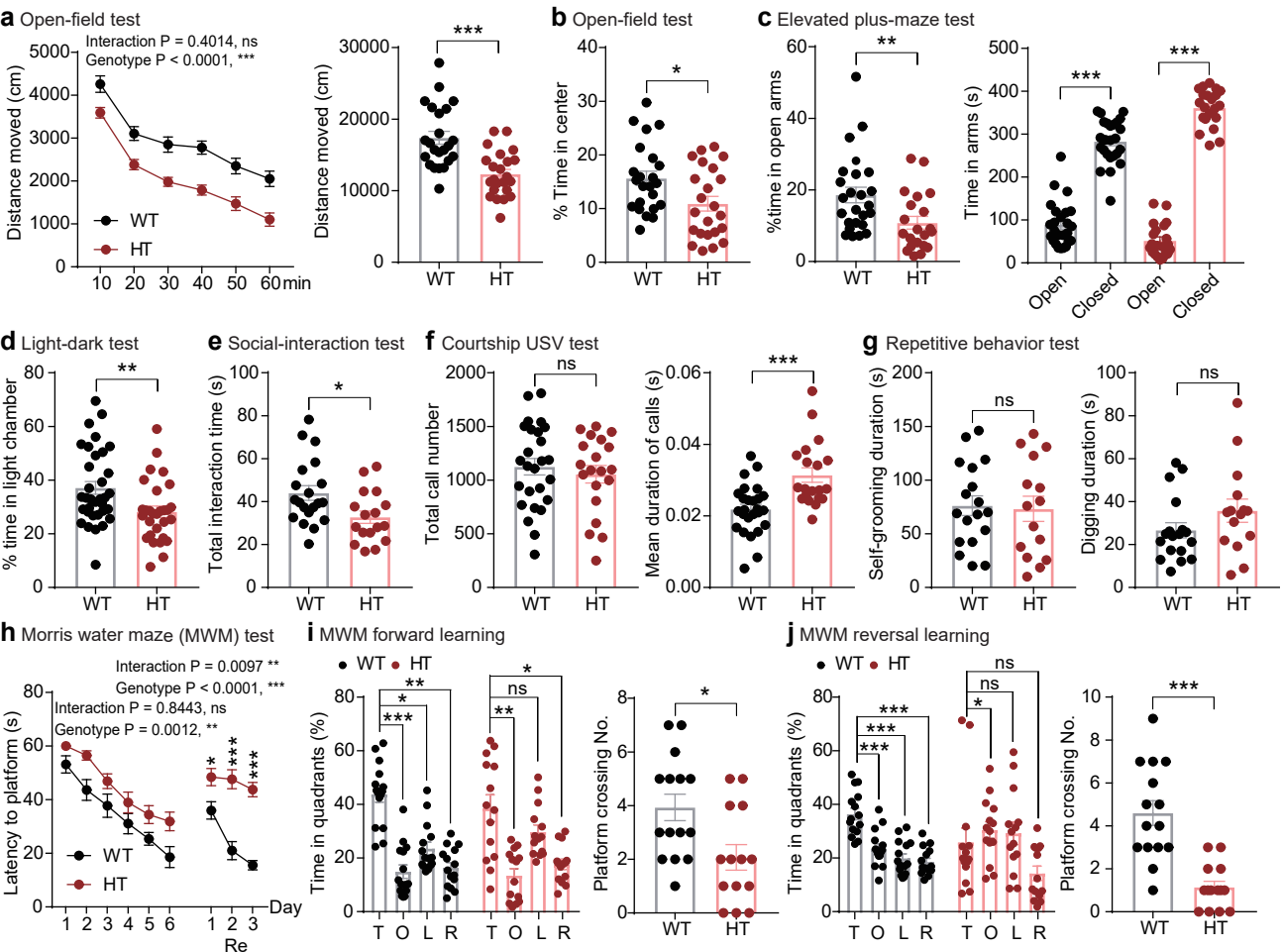

# Adult female

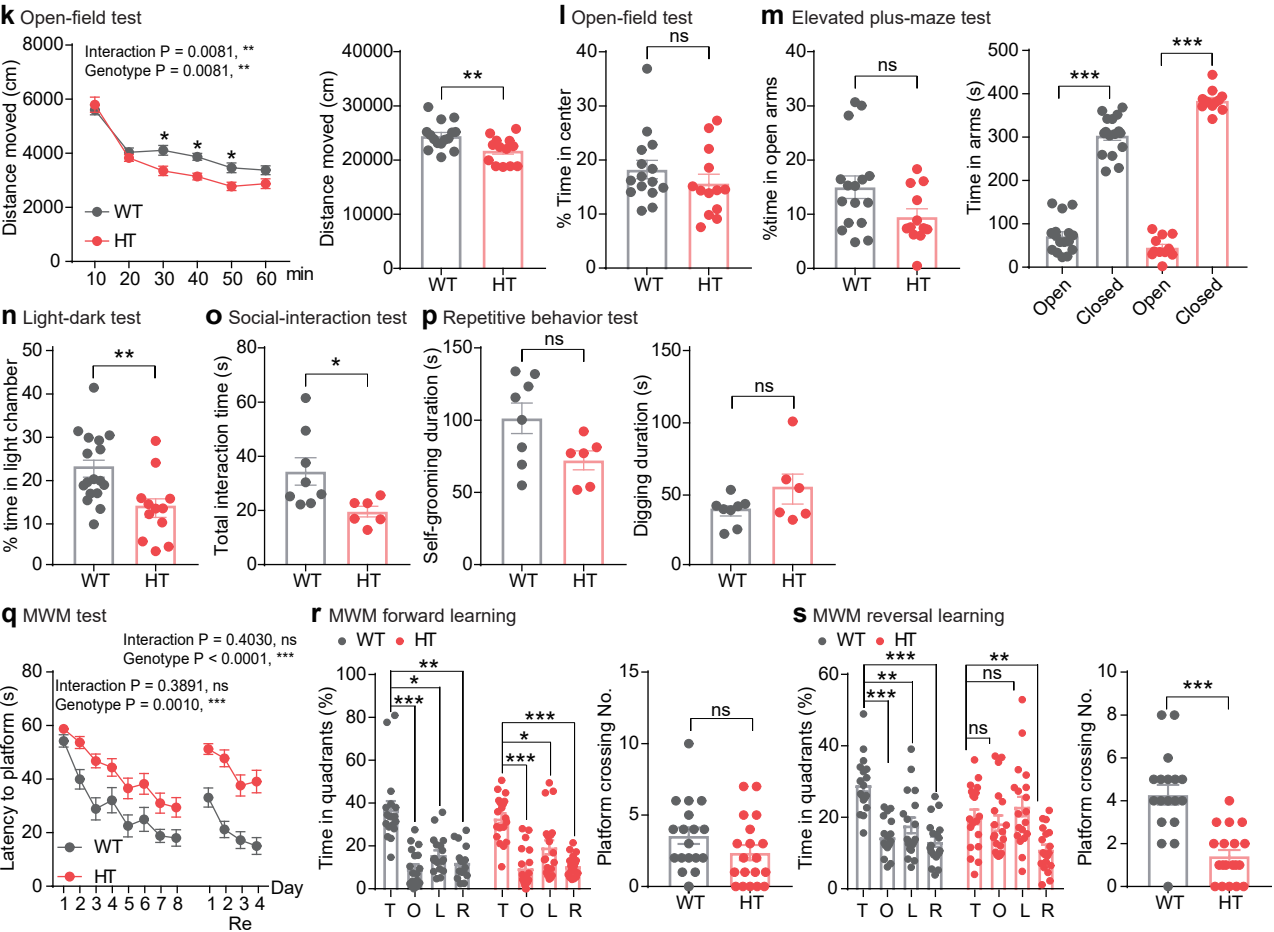

Supplement: Supplementary file 5 — Supplementary Figure 4 [file 41380_2023_2129_MOESM5_ESM.pdf]

**a** DAVID analysis of PTM-DEPP (p < 0.05; Total (1460))

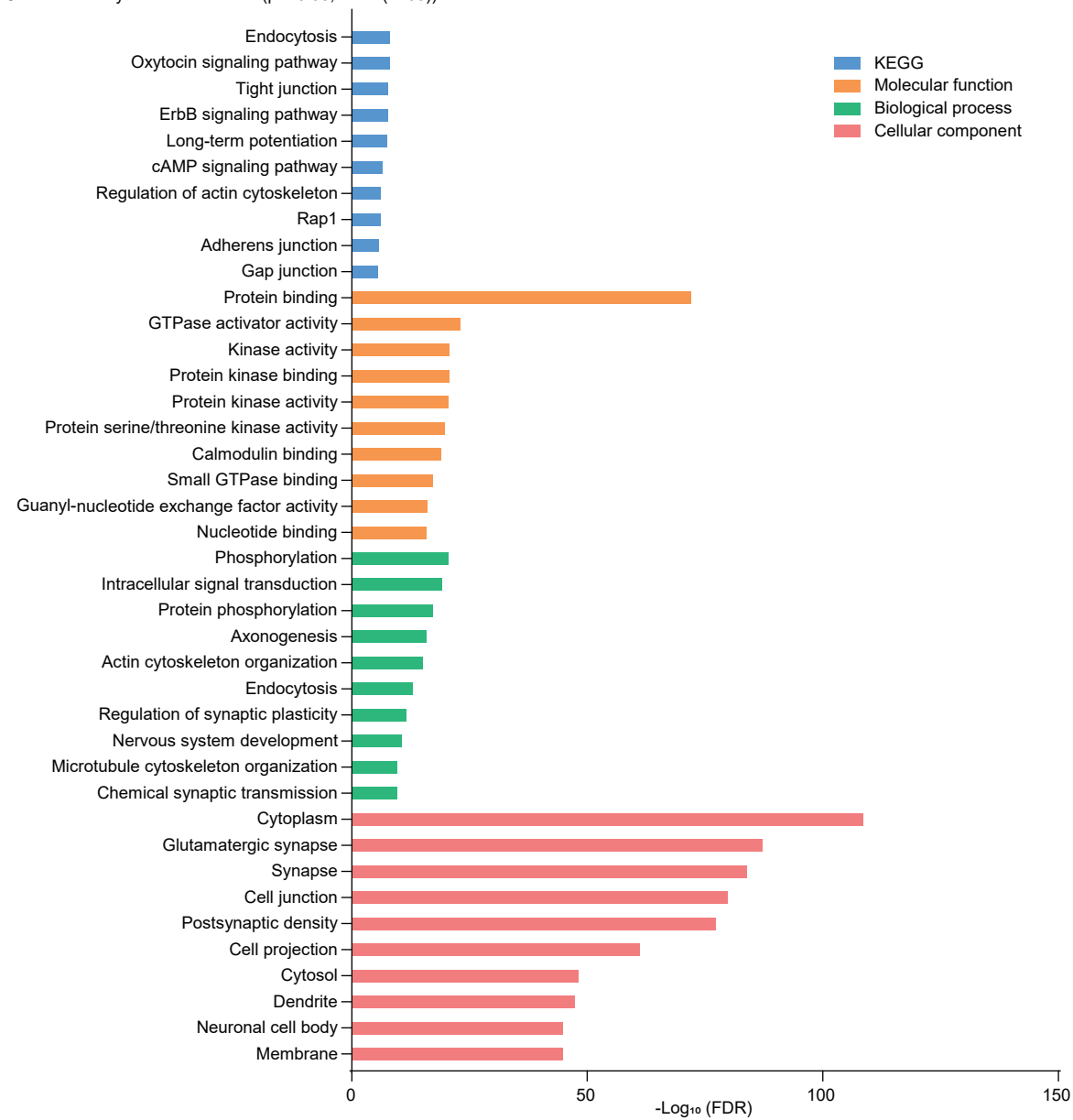

Supplement: Supplementary file 8 — Supplementary Figure 7 [file 41380_2023_2129_MOESM8_ESM.pdf]

**a** Up PTM-DEPP ( $p < 0.05 + FC > 0$ ; 824)

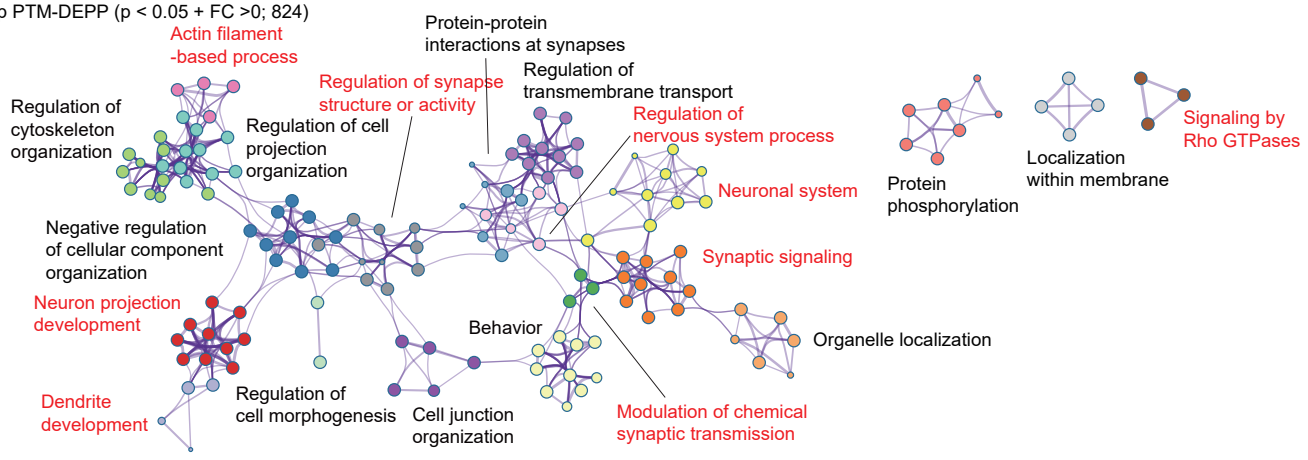

**b** Down PTM-DEPP ( $p < 0.05 + FC > 0$ ; 940)

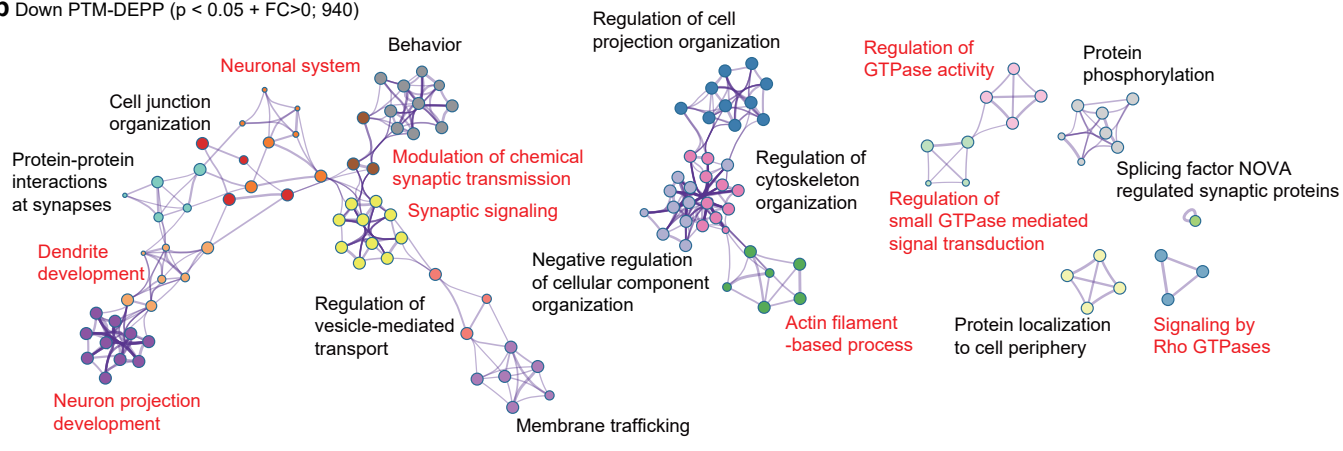

Supplement: Supplementary file 9 — Supplementary Figure 8 [file 41380_2023_2129_MOESM9_ESM.pdf]

**a** DAVID analysis of SynGO-PTM-DEPP ( $p < 0.05$  + SynGO overlap; Total (373))

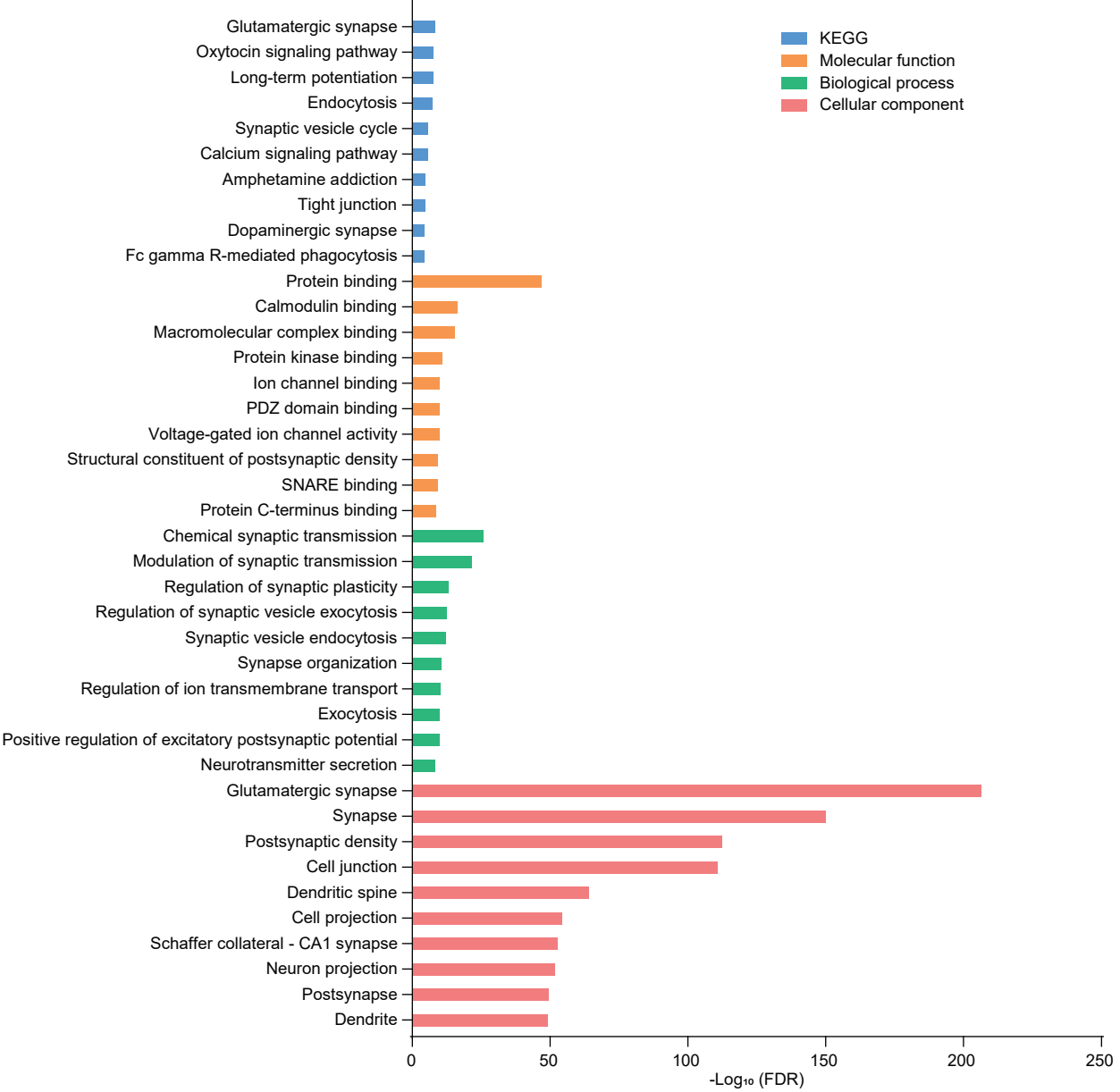

Supplement: Supplementary file 10 — Supplementary Figure 9 [file 41380_2023_2129_MOESM10_ESM.pdf]

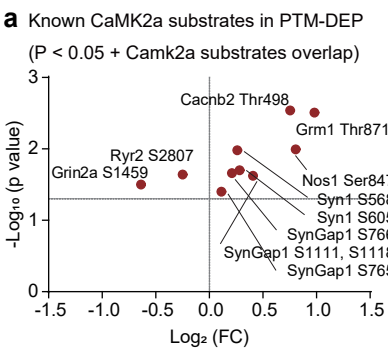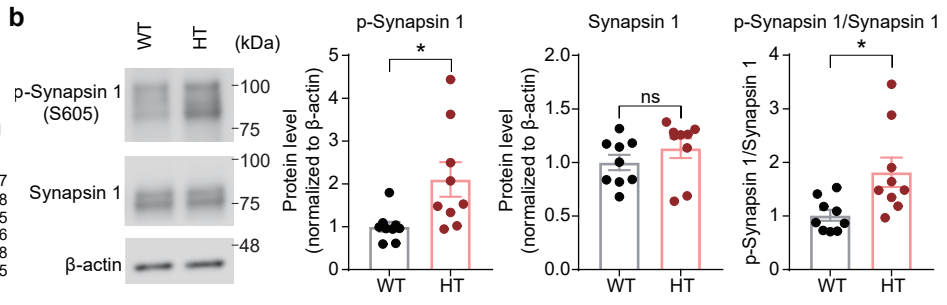

Supplement: Supplementary file 12 — Supplementary Figure 11 [file 41380_2023_2129_MOESM12_ESM.pdf]

a

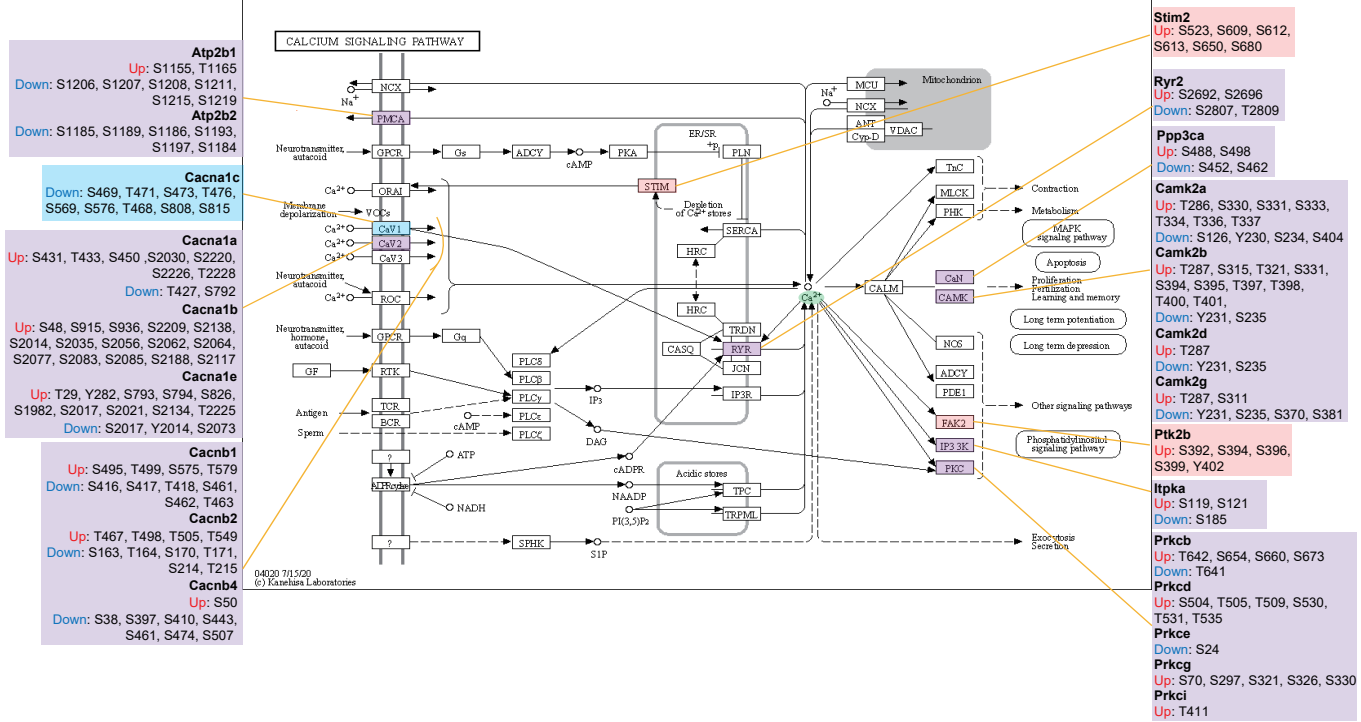

Supplement: Supplementary file 14 — Supplementary Figure 13 [file 41380_2023_2129_MOESM14_ESM.pdf]
