# Supplementary material for: Adnp-mutant mice with cognitive inflexibility, CaMKIIα hyperactivity, and synaptic plasticity deficits
Source: Mol Psychiatry. 2023 Jun 26;28(8):3548–62. doi: 10.1038/s41380-023-02129-5 (PMC10618100; doi:10.1038/s41380-023-02129-5)
Supplement: Supplementary file 4 — Supplementary Figure 3 [file 41380_2023_2129_MOESM4_ESM.pdf]

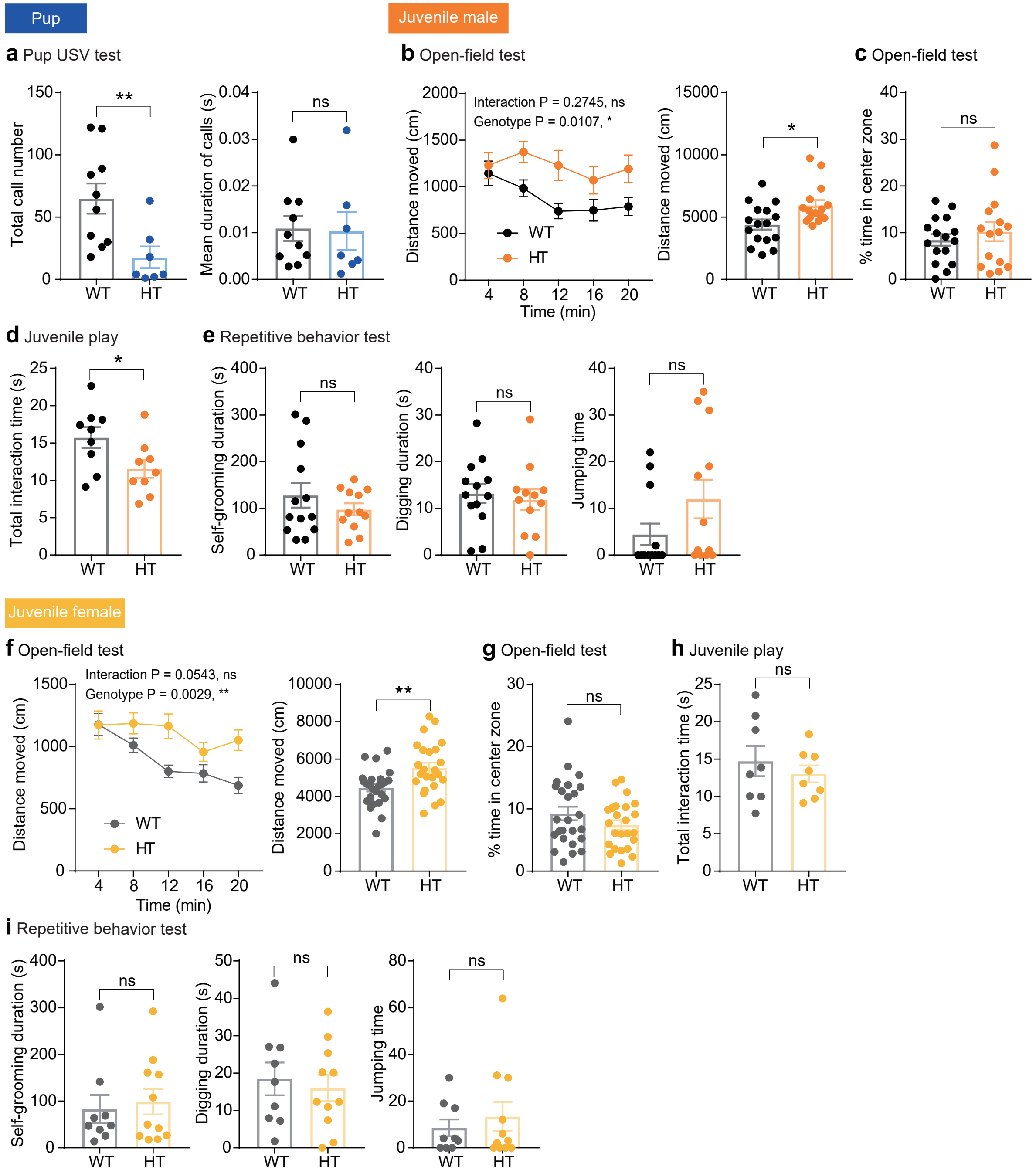

Pup
Juvenile male
a Pup USV test
Total call number
Mean duration of calls (s)
**
ns
WT
HT
b Open-field test
Interaction P = 0.2745, ns
Genotype P = 0.0107, *
Distance moved (cm)
Time (min)
c Open-field test
% time in center zone
d Juvenile play
Total interaction time (s)
e Repetitive behavior test
Self-grooming duration (s)
Digging duration (s)
Jumping time
Juvenile female
f Open-field test
Interaction P = 0.0543, ns
Genotype P = 0.0029, **
g Open-field test
h Juvenile play
i Repetitive behavior test
