# Supplementary material for: Adnp-mutant mice with cognitive inflexibility, CaMKIIα hyperactivity, and synaptic plasticity deficits
Source: Mol Psychiatry. 2023 Jun 26;28(8):3548–62. doi: 10.1038/s41380-023-02129-5 (PMC10618100; doi:10.1038/s41380-023-02129-5)
Supplement: Supplementary file 6 — Supplementary Figure 5 [file 41380_2023_2129_MOESM6_ESM.pdf]

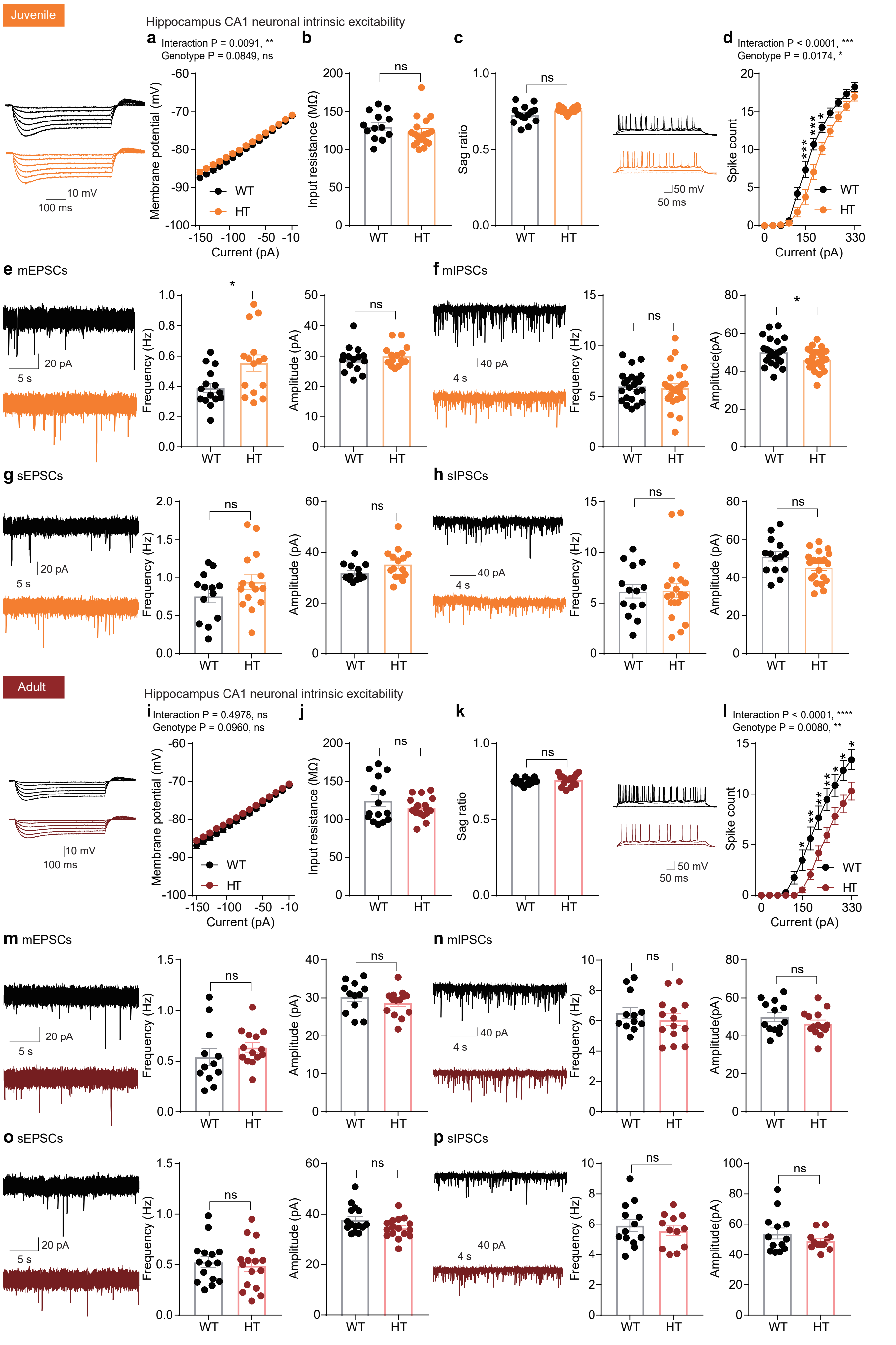
Juvenile
Hippocampus CA1 neuronal intrinsic excitability
a
Interaction P = 0.0091, **
Genotype P = 0.0849, ns
10 mV
100 ms
Membrane potential (mV)
Current (pA)
WT
HT
b
Input resistance (MΩ)
ns
c
Sag ratio
ns
50 mV
50 ms
d
Interaction P < 0.0001, ***
Genotype P = 0.0174, *
Spike count
Current (pA)
e mEPSCs
20 pA
5 s
Frequency (Hz)
Amplitude (pA)
f mIPSCs
40 pA
4 s
g sEPSCs
h sIPSCs
Adult
Hippocampus CA1 neuronal intrinsic excitability
i
Interaction P = 0.4978, ns
Genotype P = 0.0960, ns
j
k
l
Interaction P < 0.0001, ****
Genotype P = 0.0080, **
m mEPSCs
n mIPSCs
o sEPSCs
p sIPSCs
