# Supplementary material for: Adnp-mutant mice with cognitive inflexibility, CaMKIIα hyperactivity, and synaptic plasticity deficits
Source: Mol Psychiatry. 2023 Jun 26;28(8):3548–62. doi: 10.1038/s41380-023-02129-5 (PMC10618100; doi:10.1038/s41380-023-02129-5)

GSEA anlaysis of RNA-Sequencing

**a** Juvenile

| | GO terms | NES | FDR q-val |
|---|---|---|---|
| GO CC | Mitochondrial membrane part | 2.63 | 0.019 |
| | Inner mitochondrial membrane protein complex | 2.51 | 0.023 |
| | Mitochondrial protein complex | 2.42 | 0.028 |
| | Ribosome | 2.41 | 0.022 |
| | Respirasome | 2.33 | 0.029 |
| GO BP | | | |
| GO MF | Oxidoreductase activity acting on NADPH | 2.86 | 0.005 |
| | Proton transmembrane transporter activity | 2.43 | 0.041 |
| GO CC | Lysosomal lumen | -2.81 | 0.001 |
| | Extracellular matrix | -2.78 | 0.001 |
| | Collagen containing extracellular matrix | -2.45 | 0.018 |
| | Receptor complex | -2.43 | 0.016 |
| | Celll cell junction | -2.37 | 0.022 |
| GO BP | Urogenital system development | -2.86 | 0.016 |
| | Heart development | -2.82 | 0.011 |
| | Renal system development | -2.80 | 0.008 |
| | Histone methylation | -2.75 | 0.010 |
| | Aminoglycan catabolic process | -2.72 | 0.011 |
| GO MF | E box binding | -2.71 | 0.018 |
| | Voltage gated cation channel activity | -2.44 | 0.059 |
| | Metal ion transmembrane transporter activity | -2.42 | 0.045 |
| | Rac guanyl nucleotide exchange factor activity | -2.40 | 0.038 |
| | Voltage gated potassium channel activity | -2.38 | 0.033 |

**b** Adult

| | GO terms | NES | FDR q-val |
|---|---|---|---|
| GO CC | Neuron to neuron synapse | 4.64 | 0 |
| | Postsynaptic membrane | 4.57 | 0 |
| | Synaptic membrane | 4.35 | 0 |
| | Glutamatergic synapse | 4.31 | 0 |
| | Axon part | 4.21 | 0 |
| GO BP | Regulation of trans synaptic signaling | 4.89 | 0 |
| | Synapse organization | 4.73 | 0 |
| | Axon development | 4.28 | 0 |
| | Regulation of synaptic plasticity | 4.04 | 0 |
| | Regulation of cation channel activity | 4.02 | 0 |
| GO MF | Calcium ion transmembrane transporter activity | 3.45 | 0 |
| | Cation channel activity | 3.35 | 0 |
| | Gated channle activity | 3.23 | 0 |
| | Metal ion transmembrane transporter activity | 3.19 | 0 |
| | Passive transmembrane transporter activity | 3.06 | 0 |
| GO CC | Mitochondrial protein complex | -3.19 | 0 |
| | Organelle inner membrane | -3.12 | 0 |
| | Mitochondrial membrane part | -3.02 | 0 |
| | Ribosome | -2.73 | 0.001 |
| | Mitochondrial matrix | -2.70 | 0.001 |
| GO BP | Respiratory electron transporter chain | -2.97 | 0.004 |
| | Electron transport chain | -2.81 | 0.010 |
| | Mitochondrial respiratory chain complex assembly | -2.80 | 0.007 |
| | Oxidative phosphorylation | -2.65 | 0.022 |
| | ATP synthesis coupled electron transport | -2.59 | 0.032 |
| GO MF | Structural constituent of ribosome | -2.95 | 0.003 |
| | Electron transfer activity | -2.69 | 0.006 |
| | 2 Fe-2S cluster binding | -2.43 | 0.032 |

**c** Adult

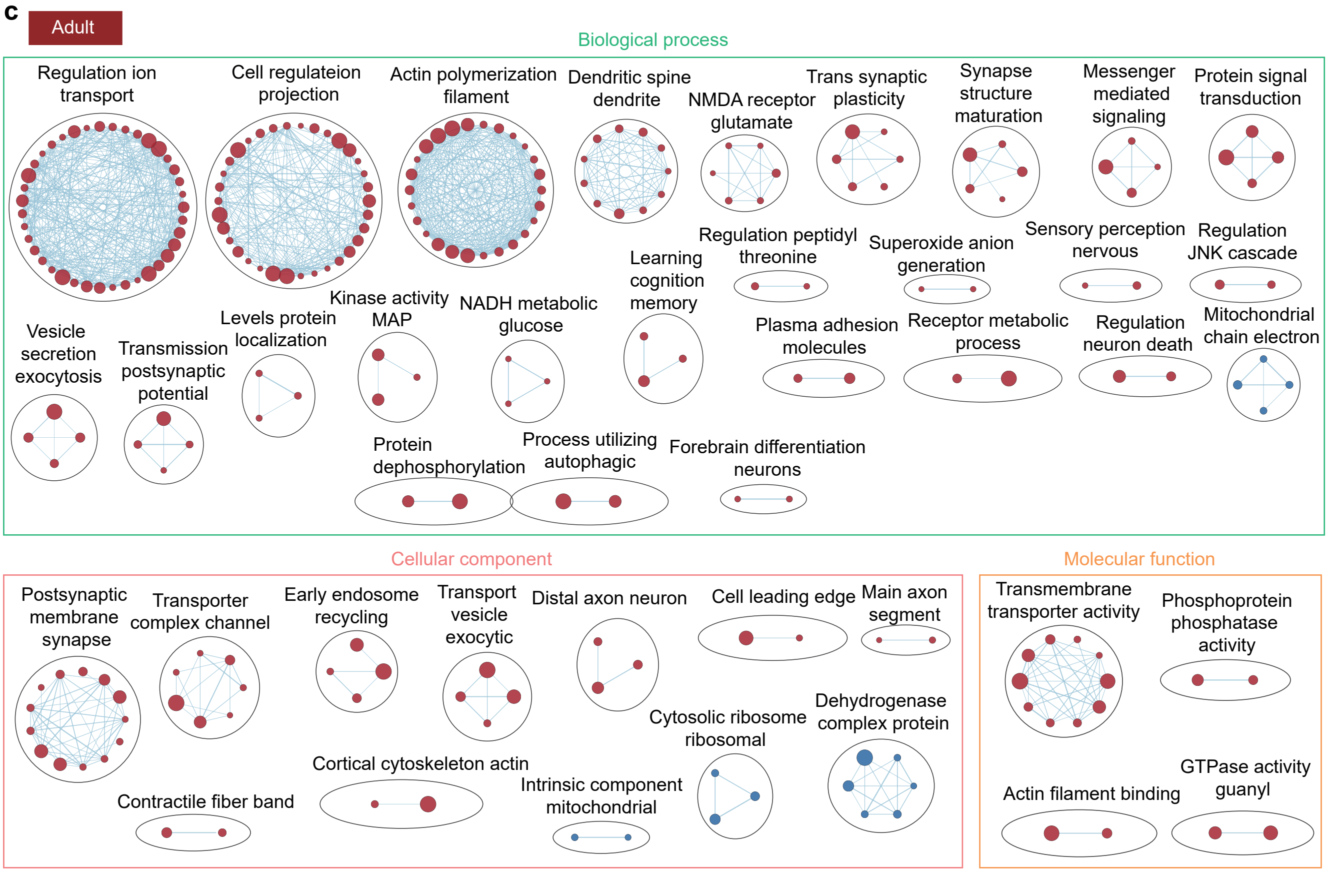


**d** FMRP targets

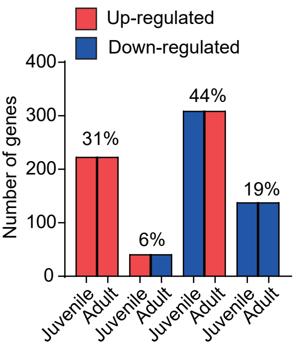


**e** FMRP targets

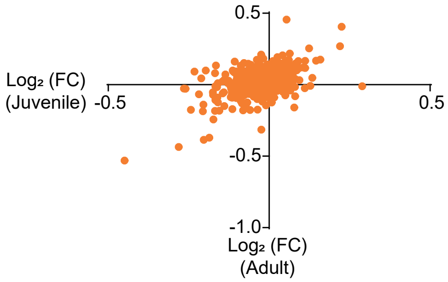

Supplement: Supplementary file 7 — Supplementary Figure 6 [file 41380_2023_2129_MOESM7_ESM.pdf]
