# Supplementary material for: Adnp-mutant mice with cognitive inflexibility, CaMKIIα hyperactivity, and synaptic plasticity deficits
Source: Mol Psychiatry. 2023 Jun 26;28(8):3548–62. doi: 10.1038/s41380-023-02129-5 (PMC10618100; doi:10.1038/s41380-023-02129-5)
Supplement: Supplementary file 11 — Supplementary Figure 10 [file 41380_2023_2129_MOESM11_ESM.pdf]

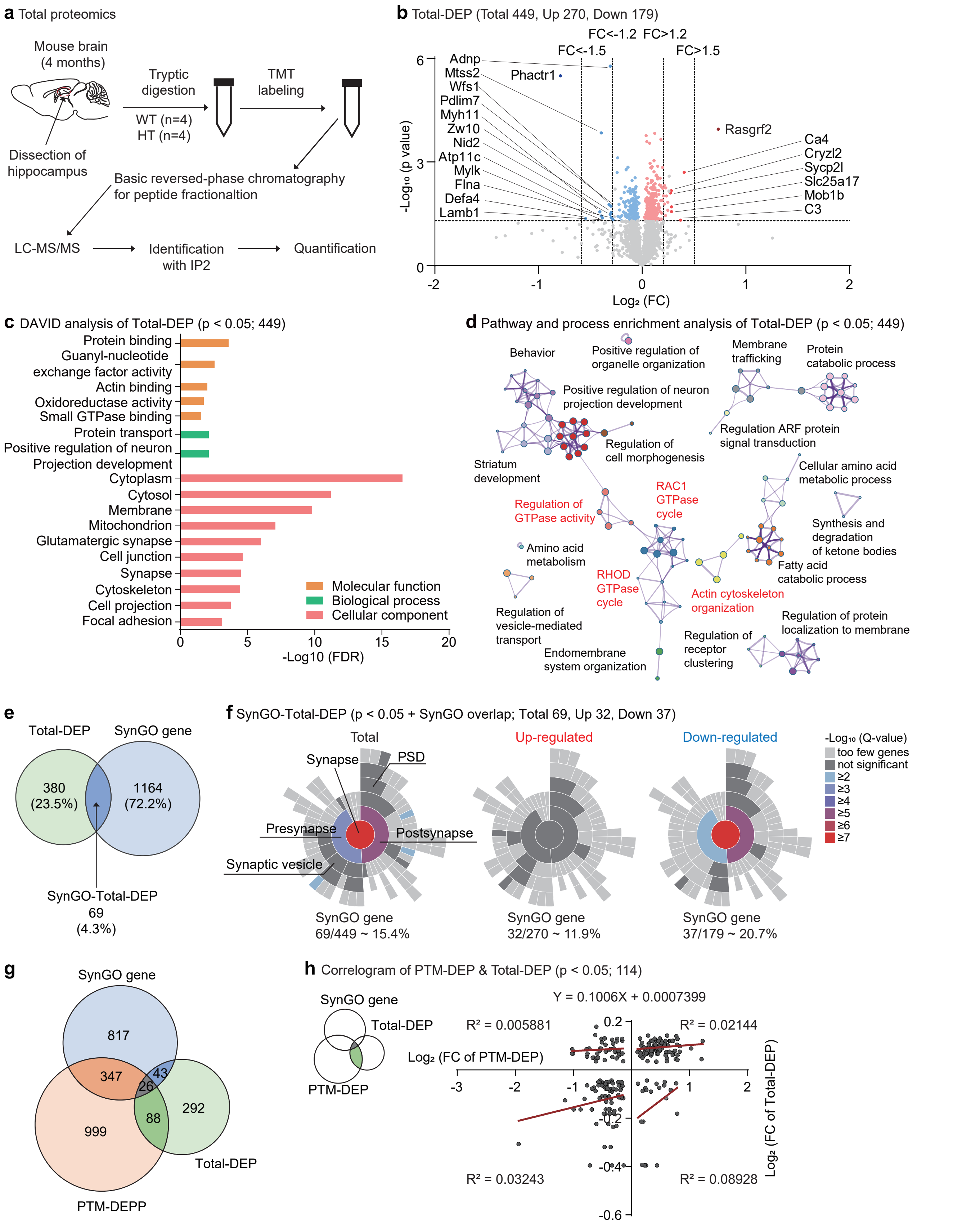

a Total proteomics
Mouse brain (4 months)
Dissection of hippocampus
Tryptic digestion
WT (n=4)
HT (n=4)
TMT labeling
Basic reversed-phase chromatography for peptide fractionaltion
LC-MS/MS
Identification with IP2
Quantification
b Total-DEP (Total 449, Up 270, Down 179)
FC<-1.2 FC>1.2
FC<-1.5
FC>1.5
Adnp
Mtss2
Wfs1
Pdlim7
Myh11
Zw10
Nid2
Atp11c
Mylk
Flna
Defa4
Lamb1
Phactr1
Rasgrf2
Ca4
Cryzl2
Sycp2l
Slc25a17
Mob1b
C3
-Log₁₀ (p value)
Log₂ (FC)
c DAVID analysis of Total-DEP (p < 0.05; 449)
Protein binding
Guanyl-nucleotide exchange factor activity
Actin binding
Oxidoreductase activity
Small GTPase binding
Protein transport
Positive regulation of neuron Projection development
Cytoplasm
Cytosol
Membrane
Mitochondrion
Glutamatergic synapse
Cell junction
Synapse
Cytoskeleton
Cell projection
Focal adhesion
Molecular function
Biological process
Cellular component
-Log10 (FDR)
d Pathway and process enrichment analysis of Total-DEP (p < 0.05; 449)
Behavior
Positive regulation of organelle organization
Membrane trafficking
Protein catabolic process
Positive regulation of neuron projection development
Regulation ARF protein signal transduction
Regulation of cell morphogenesis
Striatum development
Cellular amino acid metabolic process
RAC1 GTPase cycle
Regulation of GTPase activity
Synthesis and degradation of ketone bodies
Amino acid metabolism
Fatty acid catabolic process
RHOD GTPase cycle
Actin cytoskeleton organization
Regulation of vesicle-mediated transport
Regulation of protein localization to membrane
Regulation of receptor clustering
Endomembrane system organization
e
Total-DEP
SynGO gene
380 (23.5%)
1164 (72.2%)
SynGO-Total-DEP 69 (4.3%)
f SynGO-Total-DEP (p < 0.05 + SynGO overlap; Total 69, Up 32, Down 37)
Total
Up-regulated
Down-regulated
Synapse
PSD
Presynapse
Postsynapse
Synaptic vesicle
SynGO gene 69/449 ~ 15.4%
SynGO gene 32/270 ~ 11.9%
SynGO gene 37/179 ~ 20.7%
-Log₁₀ (Q-value)
too few genes
not significant
≥2
≥3
≥4
≥5
≥6
≥7
g
SynGO gene
817
347
43
26
88
292
999
Total-DEP
PTM-DEPP
h Correlogram of PTM-DEP & Total-DEP (p < 0.05; 114)
SynGO gene
Total-DEP
PTM-DEP
Y = 0.1006X + 0.0007399
R² = 0.005881
R² = 0.02144
R² = 0.03243
R² = 0.08928
Log₂ (FC of PTM-DEP)
Log₂ (FC of Total-DEP)
