# Supplementary material for: Adnp-mutant mice with cognitive inflexibility, CaMKIIα hyperactivity, and synaptic plasticity deficits
Source: Mol Psychiatry. 2023 Jun 26;28(8):3548–62. doi: 10.1038/s41380-023-02129-5 (PMC10618100; doi:10.1038/s41380-023-02129-5)

a

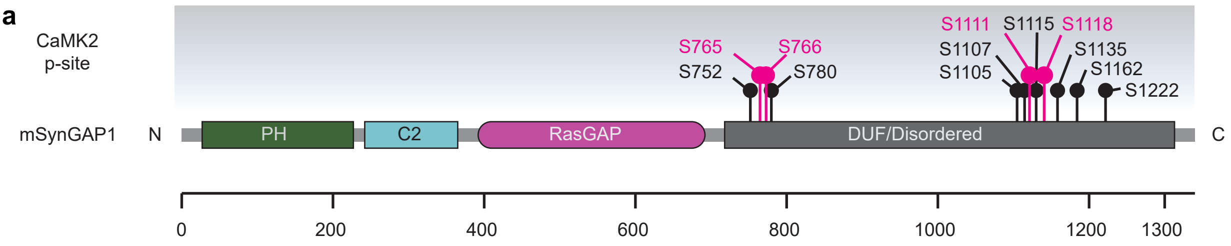

b

		PDZ1 domain			PDZ2 domain			PDZ3 domain		
		DeltaG (Kcal/mol)			DeltaG (Kcal/mol)			DeltaG (Kcal/mol)		
		Nat	SD	pS	Nat	SD	pS	Nat	SD	pS
S752	PMVLRGPSAEMQGYM	-6.6	-5.2	-4.7	-6.3	-7.0	-4.7	-7.8	-6.8	-6.2
**S765**	YMMRDLNSSIDLQSF	-9.0	-7.1	-6.0	-7.1	-8.0	-5.6	-6.9	-8.1	-6.4
**S766**	MMRDLNSSIDLQSFM	-7.0	-8.3	-4.9	-7.6	-6.5	-6.3	-9.4	-6.1	-7.5
S780	MARGLNSSMDMARLP	-8.1	-6.2	-5.6	-7.0	-8.5	-6.4	-10.0	-6.9	-4.3
S1105	PARPRQQSLSKEGSI	-6.0	-5.3	-5.0	-6.7	-7.2	-5.8	-7.8	-8.4	-5.5
S1107	RPRQQSLSKEGSIGG	-10.5	-7.6	-5.3	-7.3	-9.2	-7.4	-6.8	-7.6	-5.3
**S1111**	QSLSKEGSIGGSGGS	-6.4	-7.7	-5.7	-7.2	-7.8	-5.8	-7.9	-7.6	-6.4
S1115	KEGSIGGSGGSGGGG	-8.0	-8.5	-3.6	-8.7	-7.7	-4.8	-7.6	-7.5	-5.8
**S1118**	KEGSIGGSGGSGGGG	-8.0	-7.8	-4.5	-8.7	-6.6	-5.1	-7.6	-7.5	-6.0
S1135	PSITKQHSQTPSTLN	-7.8	-9.5	-6.7	-9.5	-9.7	-7.2	-8.1	-8.9	-7.0
S1162	VSNMPHLSADIESAH	-7.3	-7.7	-6.1	-8.4	-8.6	-6.9	-7.7	-8.8	-6.4
S1222	EYERRLLSQEEQTSk	-8.8	-5.2	-6.3	-7.2	-6.2	-7.7	-7.5	-6.3	-5.9

c **d** **e**

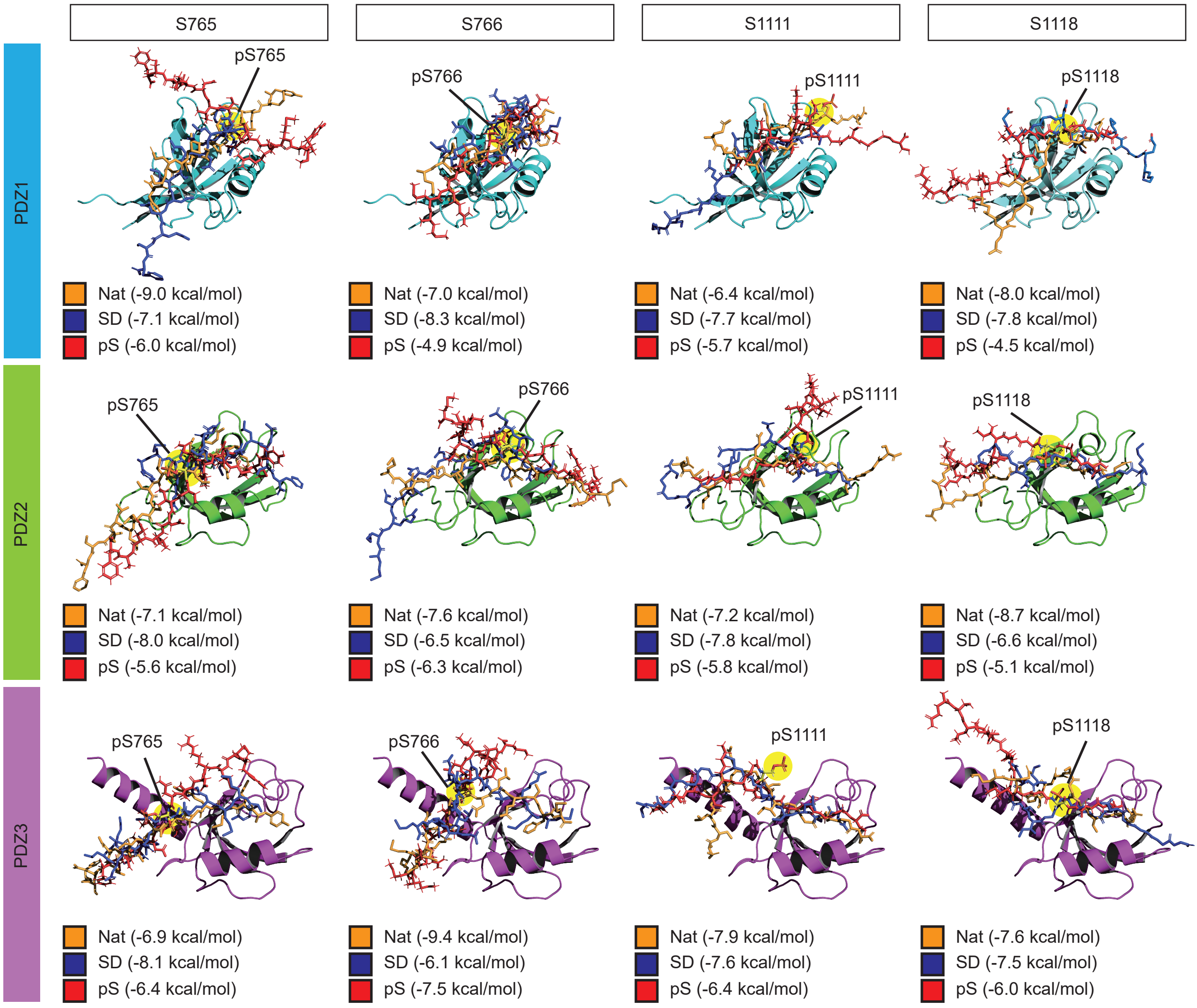

Supplement: Supplementary file 13 — Supplementary Figure 12 [file 41380_2023_2129_MOESM13_ESM.pdf]
