# Supplementary material for: Adnp-mutant mice with cognitive inflexibility, CaMKIIα hyperactivity, and synaptic plasticity deficits
Source: Mol Psychiatry. 2023 Jun 26;28(8):3548–62. doi: 10.1038/s41380-023-02129-5 (PMC10618100; doi:10.1038/s41380-023-02129-5)
Supplement: Supplementary file 15 — Supplementary Table [file 41380_2023_2129_MOESM15_ESM.zip › Supplementary Table 10.docx]

**Supplementary Table 10. Summary of the behavioral and electrophysiological results from the current study.**

|  | **Behavior domain** | **Behavior test** | **Adnp HT** | |
| --- | --- | --- | --- | --- |
|  |  |  | **Male** | **Female** |
| **Pup** | Social communication | Pup USV test | **Total call number ↓** | |
| **Juvenile** | Basal activity | Open field test | **Hyperactivity** | **Hyperactivity** |
|  | Social interaction test | Juvenile play | **Total interaction ↓** | NS |
|  | Repetitive behavior test | Home cage repetitive behavior test | NS | NS |
| **Adult** | Basal activity | Open field test | **Hypoactivity** | **Hypoactivity** |
|  | Anxiety | Open field test | **Anxiety-like behavior** | NS |
|  |  | Elevated plus maze test |  |  |
|  |  | Light-dark test |  | **Anxiety-like behavior** |
|  | Social interaction test | Direct social interaction test | **Total interaction ↓** | **Total interaction ↓** |
|  | Social communication | Courtship USV test | **Mean duration ↑** | NM |
|  | Repetitive behavior test | Home cage repetitive behavior test | NS | NS |
|  | Learning and memory | Morris water maze test | **Impaired forward learning and memory & reversal learning and memory** | **Impaired forward learning & reversal learning and memory** |

NS: Not significant, NM: Not measured

| Measurement | | Juvenile (3 weeks) | Adult (2-4 months) |
| --- | --- | --- | --- |
| Whole cell recording | Nueronal intrinsic excitability | **Spike count ↓** | **Spike count ↓** |
|  | mEPSCs | **Frequency ↑** | NS |
|  | mIPSCs | **Amplitude ↓** | NS |
|  | sEPSCs | NS | NS |
|  | sIPSCs | NS | NS |
|  | NMDA/AMPA | NS | NS |
| Field recording | Paired pulse ratio | **Slope ratio ↓** | NS |
|  | Input/output | NS | **Interaction change** |
|  | 100HZ HFS LTP | NS | **↑** |
|  | 1HZ LFS LTD | **↓** | **↓** |

NS: Not significant
