# Supplementary material for: Adnp-mutant mice with cognitive inflexibility, CaMKIIα hyperactivity, and synaptic plasticity deficits
Source: Mol Psychiatry. 2023 Jun 26;28(8):3548–62. doi: 10.1038/s41380-023-02129-5 (PMC10618100; doi:10.1038/s41380-023-02129-5)
Supplement: Supplementary file 15 — Supplementary Table [file 41380_2023_2129_MOESM15_ESM.zip › Supplementary Table 11.docx]

**Supplementary Table 11. The order of behavior experiments performed.**

| 1. The order of behavior experiments for juvenile male Adnp-HT mice | | | |
| --- | --- | --- | --- |
| Cohort1 | Open-field test | Repetitive behavior |  |
| Cohort2 | Open-field test |  |  |
| Cohort3 | Repetitive behavior |  |  |
| Cohort4 | Open-field test | Juvenile play |  |
| Cohort5 | Juvenile play |  |  |

| 2. The order of behavior experiments for juvenile female Adnp-HT mice | | | |
| --- | --- | --- | --- |
| Cohort1 | Open-field test | Repetitive behavior |  |
| Cohort2 | Open-field test |  |  |
| Cohort3 | Repetitive behavior |  |  |
| Cohort4 | Open-field test | Juvenile play |  |
| Cohort5 | Open-field test | Juvenile play |  |

| 3. The order of behavior experiments for adult male Adnp-HT mice | | | | | | |
| --- | --- | --- | --- | --- | --- | --- |
| Cohort1 | Open-field test | Elevated plus-maze | Light-dark test | Direct interaction | Repetitive behavior | Adult USV |
| Cohort2 | Elevated plus-maze | Light-dark test | Direct interaction | Repetitive behavior | Adult USV | Morris water maze |
| Cohort3 | Elevated plus-maze | Light-dark test | Direct interaction | Adult USV |  |  |
| Cohort4 | Open-field test | Light-dark test | Direct interaction |  |  |  |
| Cohort5 | Open-field test | Repetitive behavior | Morris water maze |  |  |  |

| 4. The order of behavior experiments for adult female Adnp-HT mice | | | | | | |
| --- | --- | --- | --- | --- | --- | --- |
| Cohort1 | Open-field test | Elevated plus-maze | Light-dark test | Direct interaction |  |  |
| Cohort2 | Open-field test | Elevated plus-maze | Light-dark test | Direct interaction | Repetitive behavior | Morris water maze |
| Cohort3 | Morris water maze |  |  |  |  |  |
